# Expression and purification of a single-chain Type IV restriction enzyme Eco94GmrSD and determination of its substrate preference

**DOI:** 10.1038/srep09747

**Published:** 2015-05-19

**Authors:** Xinyi He, Victoria Hull, Julie A. Thomas, Xiaoqing Fu, Sonal Gidwani, Yogesh K. Gupta, Lindsay W. Black, Shuang-yong Xu

**Affiliations:** 1 New England Biolabs, Inc., 240 County Road, Ipswich, MA 01938, USA; 2 State Key Laboratory of Microbial Metabolism, and School of Life Sciences & Biotechnology Shanghai Jiao Tong University, 1954 Huashan Road, Shanghai, 200030, China; 3 Department of Biochemistry and Molecular Biology, University of Maryland School of Medicine, 108 North Green St, Baltimore, MD 21201-1503, USA; 4 Department of Structural and Chemical Biology, Icahn School of Medicine at Mount Sinai, Box 1677, 1425 Madison Avenue, New York, NY 10029, USA

## Abstract

The first reported Type IV restriction endonuclease (REase) GmrSD consists of GmrS and GmrD subunits. In most bacteria, however, the *gmrS* and *gmrD* genes are fused together to encode a single-chain protein. The fused coding sequence for ECSTEC94C_1402 from *E. coli* strain STEC_94C was expressed in T7 Express. The protein designated as Eco94GmrSD displays modification-dependent ATP-stimulated REase activity on T4 DNA with glucosyl-5-hydroxymethyl-cytosines (glc-5hmC) and T4gt DNA with 5-hydroxymethyl-cytosines (5hmC). A C-terminal 6xHis-tagged protein was purified by two-column chromatography. The enzyme is active in Mg^2+^ and Mn^2+^ buffer. It prefers to cleave large glc-5hmC- or 5hmC-modified DNA. In phage restriction assays, Eco94GmrSD weakly restricted T4 and T4gt, whereas T4 IPI*-deficient phage (Δ*ip1*) were restricted more than 10^6^-fold, consistent with IPI* protection of *E. coli* DH10B from lethal expression of the closely homologous *E. coli* CT596 GmrSD. Eco94GmrSD is proposed to belong to the His-Asn-His (HNH)-nuclease family by the identification of a putative C-terminal REase catalytic site D507-H508-N522. Supporting this, GmrSD variants D507A, H508A, and N522A displayed no endonuclease activity. The presence of a large number of fused GmrSD homologs suggests that GmrSD is an effective phage exclusion protein that provides a mechanism to thwart T-even phage infection.

Restriction endonucleases (REases) are a diverse group of DNA-cleaving enzymes that serve to protect bacteria against phage infection or invasion of mobile genetic elements (see reviews^1^,^2^). In order to overcome attack by REases, bacteriophages evolve elaborate modifications on their genomic DNA. Bacteria, in turn, develop new enzymes that can specifically target modified DNA. Modification-dependent REases such as McrBC, McrA, Mrr, GmrSD, and PvuRts1I are loosely grouped together and referred to as Type IV REases (recent review in Ref. 3). The modified bases on DNA are N6-methyladenine (N6mA) restricted by Mrr, 5-methylcytosine (5mC) restricted by McrBC and Mrr, 5-hydroxymethylcytosine (5hmC) restricted by PvuRts1I-family enzymes and McrBC, and glucosyl-5-hydroxymethyl-cytosine (glc-5hmC) restricted by AbaSI or GmrS/GmrD enzymes^4^,^5^,^6^,^7^,^8^,^9^,^10^. The first characterized GmrSD enzyme was found in *E. coli* strain CT596 and encoded by two adjacent genes *gmrS* and *gmrD*^6^,^7^. The GmrS and GmrD subunits were separately expressed as intein and chitin-binding domain (CBD) fusion proteins and cleaved off by intein cleavage. The reconstituted enzyme is active and specific for glc-5hmC-modified DNA, but it has poor activity on 5hmC- or 5mC-modified DNA. The reaction buffer for the reconstituted enzyme included UTP, Ca^2+^, and Mg^2+^^6^. It has been reported that T-even phages encapsidate a diverse set of internal proteins encoded at the *ip1* locus that function to counteract the GmrSD nuclease activity during DNA injection^7^,^11^ (*ip1*, T4 phage inhibitor gene encoding IPI protein that is processed into encapsidated IPI* protein). Interestingly, a close homolog (UTI89_C2960 or UT enzyme) found in *E. coli* O18 K1 H7 UTI89 is a fused single-chain enzyme; the UT enzyme is insensitive to IPI* inhibition due to its altered amino acid (aa) sequence and specificity, but it does not restrict either T4 IPI*-deficient or wild-type (WT) T4 phage although it restricts many other T even-like phages such as T2 and T6^12^. The enzymatic tools that differentially cleave 5hmC DNA are limited, since McrBC- and MspJI-family enzymes cleave both 5hmC and 5mC-modified DNA^13^,^14^. Structural studies that could determine the interactions that occur between GmrSD and its inhibitor protein, IPI*, have been hampered by the poor expression of the two chain GmrS/GmrD enzyme.

The flux of sequenced bacterial genomes has revealed that there are many GmrSD homologs in proteobacterial genomes. As with the UTI89_C2960 protein, in these homologs the *gmrS* and *gmrD* genes are fused together to form a single gene, which may encode a single-chain GmrSD enzyme. The goal of this work was to evaluate the endonuclease activity of such a single-chain GmrSD homolog found in the genome of *E. coli* strain STEC_94C and to develop methods for simple purification of the target protein. In addition, we studied the metal ion requirement and preferred substrate size for Eco94GmrSD, and identified a potential endonuclease catalytic site (a conserved nuclease motif Asp-His-Asn (D-H-N) in its C-terminus). We found that the single-chain enzyme is capable of cleaving 5hmC and glc-5hmC DNA in vitro. This property differs from the two-chain GmrS/GmrD enzyme complex that only cleaved glc-5hmC DNA. However, despite this difference in in vitro substrate sensitivity we found that the phage restriction activity of Eco94GmrSD is very similar to that of the two-chain GmrS/GmrD: Eco94GmrSD only weakly restricted WT T4 and T4gt (deficient in α-, β-glucosyltransferase (gt) phages), but strongly restricted T4Δ*ip1* phage (about a million fold). The possible involvement of GmrSD-like enzyme in the bacterial immigration control region (ICR) is also discussed.

## Results

The hypothetical protein ECSTEC94C_1402 (GenBank accession #: WP_000834395) from *E. coli* STEC_94C has 629 amino acid (aa) residues. It displays 93% aa sequence identity to the GmrSD fusion protein found in *E. coli* UTI89 (UTI89_C2960, EcoUTI89GmrSD or UT enzyme) (see sequence alignment in Supplementary Fig. S1). The ECSTEC94C_1402 gene is located on a 41.5 kb region of the ECSTEC94C genome diagnosed by “Phast” to be a prophage most similar to *Shigella* phage SfII (NC_021857). This similarity is mostly over the first 17 kb of the SfII genome (97% by blastn analysis), a region that encodes the major morphogenesis genes, although there are other shorter homologous regions. Notably, the Eco94GmrSD gene is immediately downstream of the genes predicted to form the phage tail fibers that are responsible for host adsorption. This is a morphogenic region known to evolve rapidly to adapt to changes in host cell receptors. Notably, *Shigella* phage SfII does not encode a GmrSD homolog at this position (or elsewhere). We hypothesize that the GmrSD gene being prophage-borne indicates it has some role in an evolutionary arms race between phage and host, supported by its in vitro and in vivo restriction properties (see below). EcoCT596GmrSD (CT enzyme) was also encoded by a curable prophage that restricted T4*ip1*^−^ and rII mutants.

Three major differences were found when Eco94GmrSD sequence was compared to the prototype EcoCT596GmrS/GmrD as shown in Supplementary Fig. S1: 1) Eco94GmrSD lacks 3-aa residues Ser97-Leu98-Ala99; 2) Eco94GmrSD carries one additional amino acid difference (Arg313 in Eco94GmrSD vs Gln313 in the two-chain CT enzyme); 3) Eco94GmrSD contains 84-aa residues as a connector of the GmrS and GmrD subunits, which fused two subunits into a single-chain peptide. Recently, the gene sequence encoding the CT enzyme has been resequenced and updated as a single gene that restricts glc-5hmC containing T-even phages as cloned in a pBeloBac11 (single copy vector)(Genbank ID: AF493796_1). It is now apparent that the two-chain GmrSD originally cloned had suffered mutational events, but still retained activity. Subcloning of the *ecoCT596gmrSD* gene into higher copy plasmids was enabled by introduction of a stop codon which caused a truncation product GmrS, and re-initiation product GmrD with a small deletion between the *gmrS* and *gmrD* genes (see below)^6^.

We used two expression strategies to express ECSTEC94C_1402. One strategy was to clone its ORF in pET21b in fusion with a C-terminal 6xHis tag (Eco94GrmSD-6xHis) and purification through a nickel-NTA agarose column. Another method was to clone its ORF in fusion with an intein and CBD, the same strategy proved successful in expression of the two-chain GmrS/GmrD originally cloned from *E. coli* strain CT596 (see Supplementary information). The Eco94GrmSD enzyme purified by two different methods (6xHis-tagged protein via nickel column or intein-CBD tagged protein via chitin column) share nearly identical enzyme properties except that the His-tagged enzyme displays higher specific activity (see below).

### Expression and purification of 6xHis-tagged single-chain GmrSD (GmrSD-6xHis) and endonuclease activity assay

**Figure 1:**
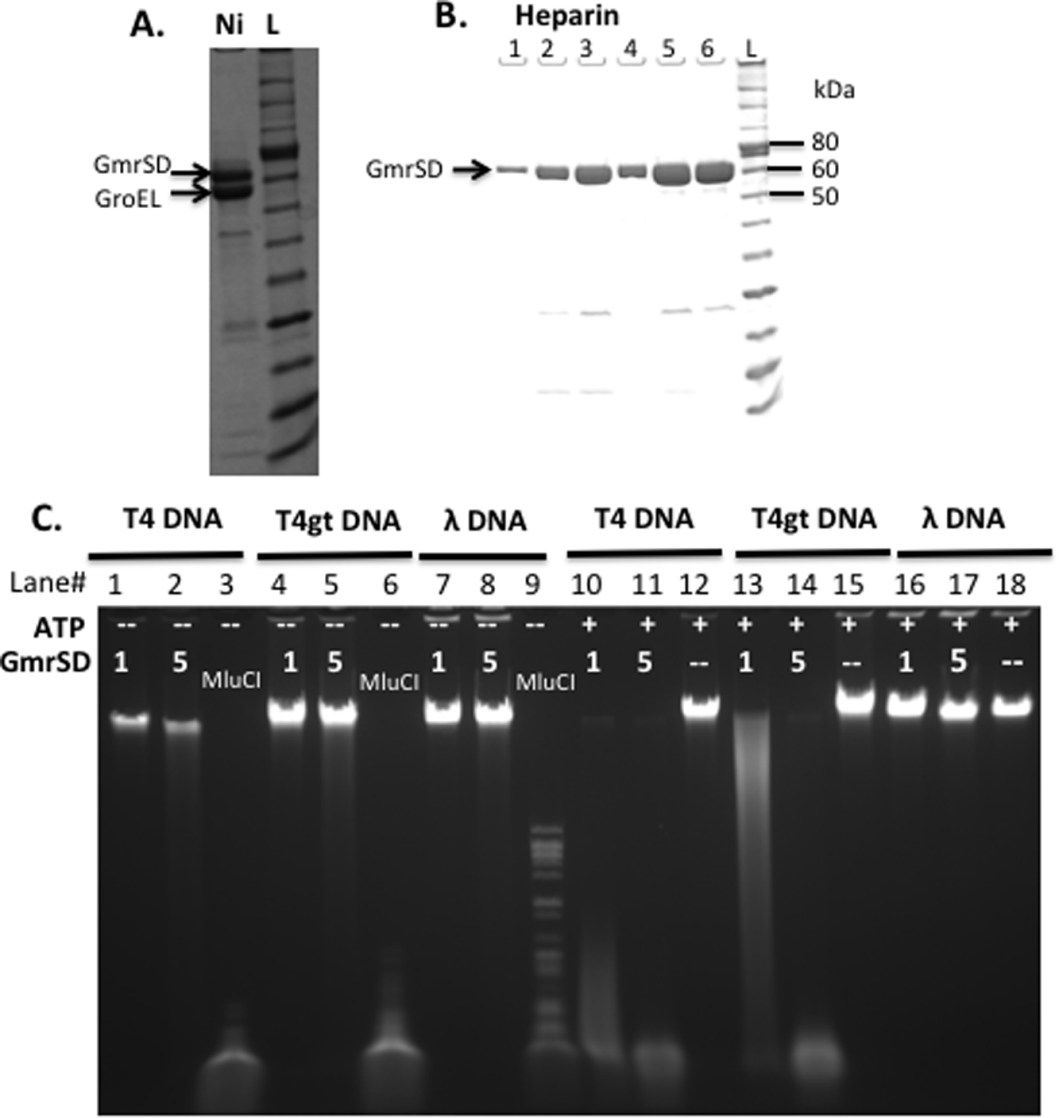
SDS-PAGE analysis of purified Eco94GrmSD-6xHis protein and agarose gel analysis of endonuclease activity assay. (A). Purified C-terminal 6xHis-tagged GrmSD from a nickel column (Ni). The enzyme was purified from cell lysate of T7 Express [pET21b-*gmrSD*, pGro7]. L, protein ladder. (B). SDS-PAGE analysis of purified GmrSD fractions from a heparin column. (C). Digestion of T4 (glc-5hmC), T4gt (5hmC) and λ DNA (Dam^+^Dcm^+^) by purified GmrSD-6xHis in the presence (+) or absence (−) of 1 mM ATP. Lanes 3, 6, and 9, MluCI digested DNAs. One μl or 5 μl of GmrSD (~0.5 μg/μl, 0.14 or 0.70 μM) was used to digest 1 μg DNA in buffer 2 plus or minus 1 mM ATP.

In one expression strategy we cloned the single-chain *eco94gmrSD* gene into pET21b and purified it with a C-terminal 6xHis-tag (GmrSD-6xHis). This protein was insoluble if IPTG-induction was carried out at 37°C, however it expressed well at 16°C to 18°C in co-overexpression of GroEL/GroES protein from a compatible plasmid (data not shown). GmrSD-6xHis protein was purified by nickel-NTA agarose column chromatography, and further purified via a heparin column. Most of the GroEL protein was removed by the second step. Fig. 1 shows the partially purified GmrSD-6xHis enzyme (pooled fractions from heparin for activity assay) and its low enzyme activity on T4 (panel C, lanes 1–2), T4gt (lanes 4–5), and λ DNA (lanes 7–8). The endonuclease activity was strongly stimulated by addition of 1 mM ATP in digestion of T4 and T4gt DNA (lanes 10–11, 13–14); poor activity was detected on λ DNA (Dam^+^ Dcm^+^). In a control experiment, T4, T4gt, and λ DNAs were digested by MluCI (AATT) whose activity was not affected by cytosine modifications. The specific activity of the purified enzyme was estimated to be ~500 units/mg protein on T4 DNA (see unit definition in Methods). The final protein yield was estimated at 4 mg/L of IPTG/arabinose-induced cells. It appeared that the GmrSD enzyme displayed lower cleavage activity on T4gt DNA compared to T4 (less than 2-fold difference).

### Metal ion and dithiothreitol (DTT) requirement for GmrSD endonuclease activity

**Figure 2:**
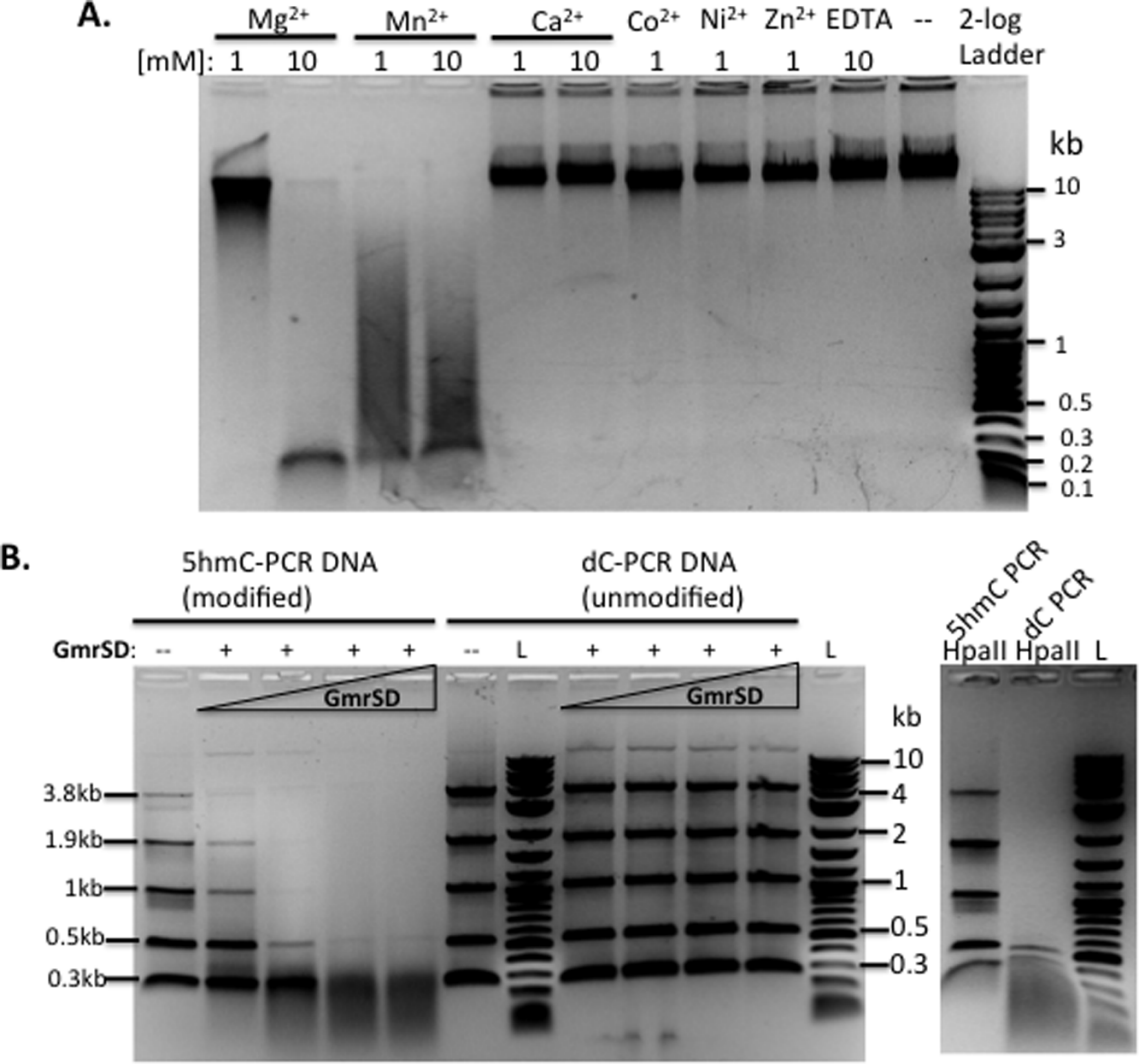
Determination of divalent cation cofactor requirement and DNA substrate preference for GmrSD digestion. (A). Metal ion cofactor requirement for GmrSD digestion. Divalent cations or EDTA are indicated on top of each lane. (B). Substrate preference and optimal substrate size for GmrSD digestion. PCR DNA substrates containing 5hmC or regular dC were generated by PCR using pBR322 template and digested by GmrSD endonuclease in the presence of 1 mM ATP in NEB buffer 2. The same DNA substrates were also digested by HpaII (CCGG) in NEB buffer 4 (to confirm modified DNA).

The purified Eco94GmrSD was tested for activity on T4 DNA in a basic buffer (50 mM NaCl, 10 mM Tris-HCl, pH 7.5, 1 mM DTT) supplemented with different divalent cations. Eco94GmrSD was active in digestion of T4 DNA when the basic buffer was supplemented with Mg^2+^ or Mn^2+^ (Fig. 2A, lanes 1–4). It was interesting to note that metal ions can modulate the relative endonuclease activity. At 1 mM of divalent cation, the enzyme is more active in the presence of Mn^2+^ than Mg^2+^. At 10 mM of the metal ion, GmrSD is more active in Mg^2+^ than Mn^2+^. The free Mg^2+^ concentration in *E. coli* cells was estimated at 1 to 2 mM^15^,^16^. The intracellular concentration of Mn^2+^ in bacteria was estimated at μM range (www.bionumbers.hms.harvard.edu). The Eco94GmrSD enzyme shows poor activity with other metal ions such as Ca^2+^, Co^2+^, Ni^2+^, or Zn^2+^ (Fig. 2A, lanes 5–9). We also compared GmrSD endonuclease activity in 10 μM, 0.1 and 1 mM of Co^2+^, Ni^2+^, or Zn^2+^ (since high concentration of transition metal ions may inhibit activity). Eco94GmrSD displayed very low activity in 0.1 mM Co^2+^ or Zn^2+^ (data not shown). GmrSD nuclease activity on T4 DNA was clearly detected in Mn^2+^ buffer (optimal concentration at 1 mM MnCl_2_). But this low nuclease activity was independent of ATP cofactor (see Supplementary Fig. S2A, lanes 1–4). It was somewhat unexpected that addition of 1 mM ATP could inhibit GmrSD activity in Mn^2+^ buffer (lanes 6–8). In a control digestion, GmrSD degraded T4 DNA into small fragments (100–300 bp) in NEB buffer 2 and 4 with 10 mM Mg^2+^. It was puzzling that the same ATP cofactor could have a positive stimulatory effect on GmrSD nuclease activity in Mg^2+^ buffer, but it exerts a negative inhibitory effect in Mn^2+^ buffer (at 0.1 to 0.5 mM). It is well known that HNH-family endonucleases are more promiscuous in metal ion cofactor requirement for catalytic activity^17^,^18^,^19^. Perhaps the negatively regulatory loop by ATP provides a safeguard to GmrSD star activity on unmodified DNA when GmrSD enzyme is “accidently” bound by Mn^2+^ ions. To see whether GmrSD enzyme displays any nuclease activity on λ DNA in Mn^2+^ buffer, λ DNA was digested by GmrSD in the absence or presence of 1 mM ATP. Supplementary Fig. S2B shows that GmrSD caused some λ DNA smearing as an indication of low nuclease activity. The supplement of 1 mM ATP appeared to inhibit nuclease activity at 0.1 to 0.5 mM Mn^2+^. In a control digestion, GmrSD enzyme shows no smearing in NEB buffer 2 and a low level of smearing in buffer 4. We speculate that GmrSD enzyme displays relaxed specificity (star activity) in Mn^2+^ buffer (since it partially cleaved non-glc-5hmC or non-5hmC DNA). This star activity is consistent with the observation that GmrSD over-expression in a RecA-deficient *E. coli* host was quite toxic (see below), probably caused by dsDNA breaks at star sites and the lack of RecA-mediated DNA recombination and repair.

Eco94GmrSD enzyme gradually loses activity during storage at −20°C, however, its activity can be restored by addition of fresh DTT (data not shown). There are seven Cys residues in Eco94GmrSD enzyme and presumably oxidation of these Cys residues may contribute to lower activity during storage. The optimal temperature for Eco94GmrSD activity was determined to be 37°C (see Supplementary Fig. S3).

### Preferred substrate and substrate size for the single-chain Eco94GmrSD

To study the substrate size preference we used PCR products that contain 5hmC incorporated during PCR by including 5hm-dCTP in PCR reactions. PCR products (3.8, 1.9, 1.0, 0.5, and 0.3 kb) containing 5hmC or unmodified dC were purified by spin columns and digested with Eco94GmrSD. 5hmC-modified PCR DNA substrates (3.8 kb, 1.9 kb, 1.0 kb) were efficiently digested; while modified PCR products in 0.3 and 0.5 kb were cleaved with reduced efficiency (Fig. 2B). PCR products (same sizes) with regular dC were poorly digested by Eco94GmrSD at the same enzyme concentration tested (Fig. 2B). This result is consistent with the substrate preference for modified DNA (T4gt) shown in Fig. 1. In a control experiment, 5hmC-modified PCR substrates were resistant to HpaII digestion and PCR DNAs with unmodified cytosine were digested by HpaII (Fig. 2B, right panel). A 60-bp PCR fragment containing 5hmC-N20-G (two 5hmC on the opposite strands separated by 20 bp) was partially cleaved by Eco94GmrSD; but the 60mer with 5hmC-N10-G (two 5hmC on the opposite strands separated by 10 bp) was not cleaved (Supplementary Fig. S4). We also cloned and sequenced some GmrSD cleavage products of T4gt and determined the cut sites (see Supplementary information and Table S1). The common feature of these cut sites was 5hmC N(17–23) G (two 5hmC on the opposite strands separated by 17–23 bp), where cleavage takes place mostly at the symmetric sites 5hmC N(9–11)↓N(9–11)G. Sequencing of more cleavage products are required to pinpoint the substrate preference and the effect of flanking sequence on cleavage efficiency of the modified 5hmC-containing DNA.

### NTP- and dNTP-stimulated GmrSD endonuclease activity

**Figure 3:**
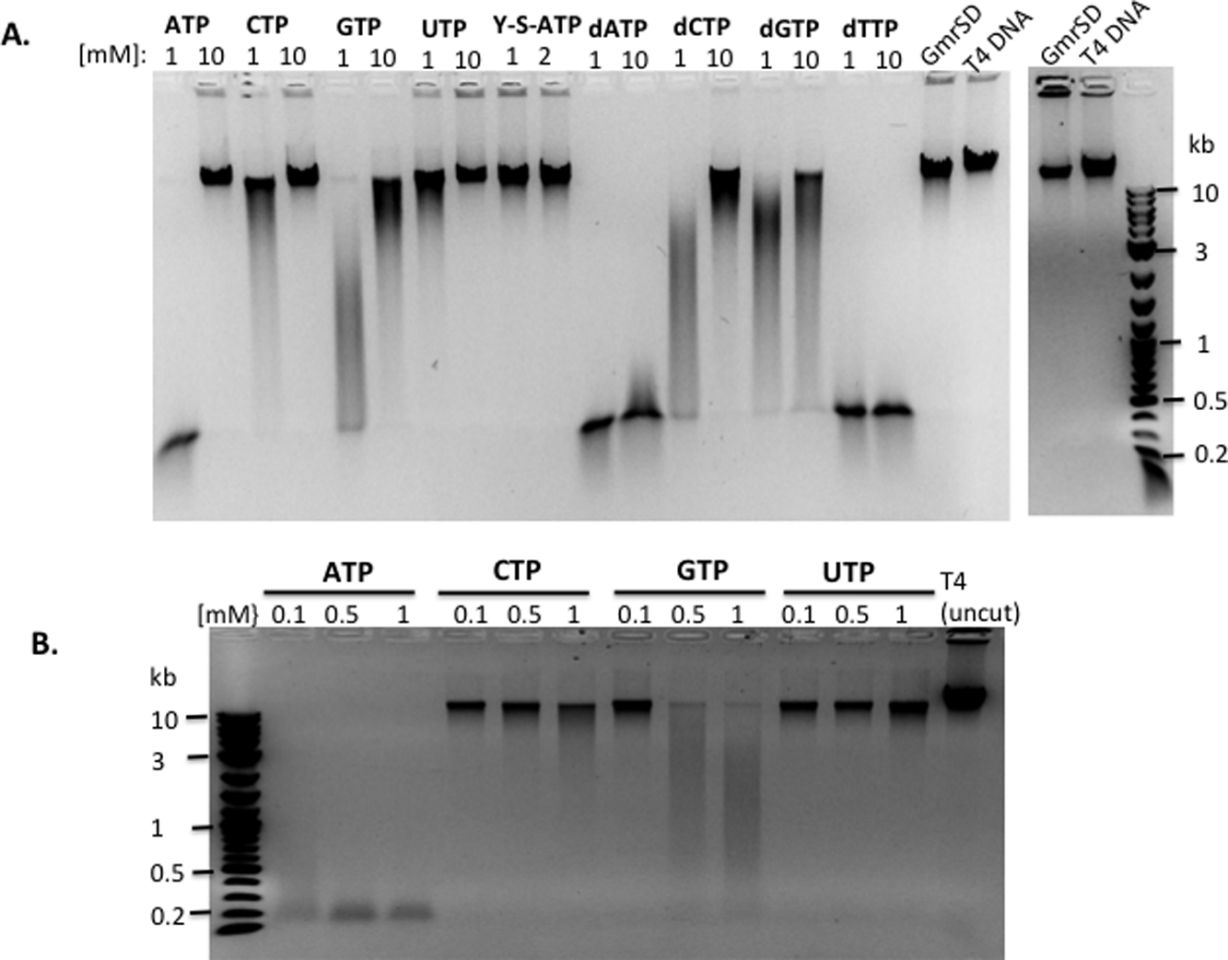
Stimulation of GmrSD endonuclease activity by supplement of NTP or dNTP in digestion of T4 DNA. No stimulator effect on enzyme activity was detected by supplement of non-hydrolysable γ–S-ATP. NTP or dNTP concentrations were indicated on top of each lane.

In the previously published report, NTP stimulated the CT enzyme activity^6^. Therefore, we examined the endonuclease activity in the presence of NTP, dNTP, or non-hydrolysable γ-S-ATP. Fig. 3A and 3B show strong stimulation of endonuclease activity by addition of 0.1 to 1 mM ATP (but higher concentration of ATP at 10 mM inhibits activity). Stimulation of activity was also detected at 2 mM ATP concentration (data not shown). Supplement of 0.1, 0.5, and 1 mM CTP or UTP had a minimal effect. Addition of GTP (0.5–1 mM) also had a moderate effect on enzyme activity. Supplement of dATP (1–10 mM), or dTTP (1–10 mM) also strongly stimulated the endonuclease activity, while dCTP and dGTP have moderate effect. But addition of non-hydrolysable γ-S-ATP (1–2 mM) had no stimulatory effect on enzyme activity. We have not directly measured NTP hydrolysis in GmrSD cleavage reactions.

### Site-directed mutagenesis of a putative catalytic site in the C-terminal domain

**Figure 4:**
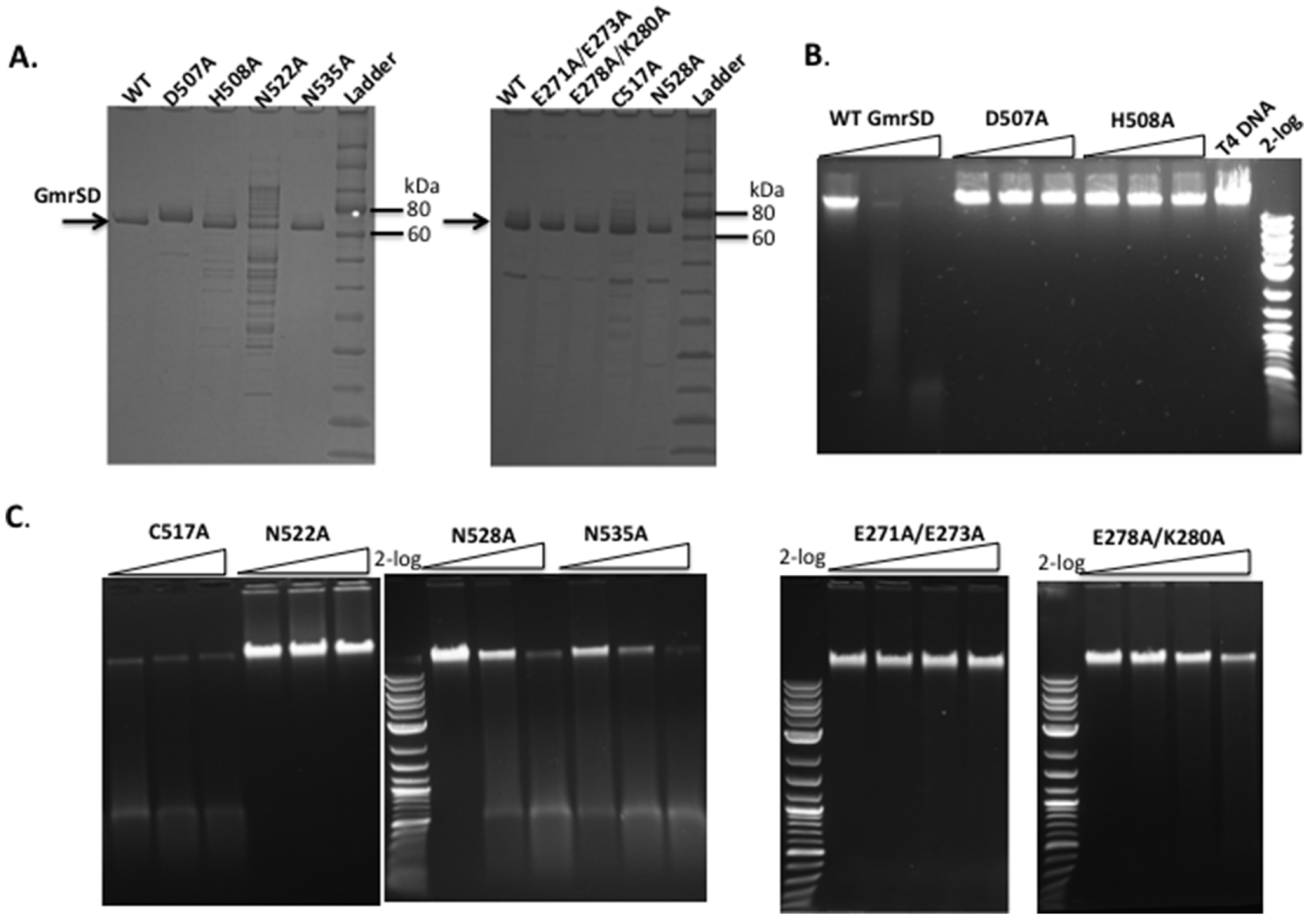
Analysis of partially purified WT Eco94GmrSD and mutant proteins D507A, H508A, C517A, N522A, N528A, N535A, E271A/E273A, E278A/K280A on SDS-PAGE and endonuclease activity assays for the mutant enzymes on T4 DNA. (A). SDS-PAGE analysis of WT and mutant proteins. Left panel: WT and mutant proteins (D507A, H508A, N522A, and N535A) purified by nickel-NTA agarose and heparin HP columns (purified D507A showed aberrant migration). Right panel: WT, E271A/E273A, E278A/K280A, C517A and N528A proteins. (B and C). Endonuclease activity assay for WT and GmrSD variants D507A, H508A, C517A, N522A, N528A, N535A on T4 DNA. The amount of input protein was 0.5 μg, 1 μg, and 2 μg, respectively in digestion of 1 μg T4 DNA. For the double mutants E271A/E273A and E278A/K280A, the amount of input protein was 0.5 μg, 1 μg, 1.5 μg and 2 μg, respectively in digestion of 1 μg T4 DNA.

In a protein homology search, Eco94GmrSD had a weak hit with His-metal finger nuclease family (conserved amino acid residues DHxxP). The putative endonuclease active site residues located near the C-terminus are D507-H508-N522-(N528-N535) with additional Asn/Gln/Lys residues in close proximity (conforming to DH-N-N catalytic site). The HNH (HNK or HNN) motif is found in Colicin nucleases, homing endonucleases, DNA repair enzymes, REases, DNA nicking enzyme, transposase, type II intron-encoded reverse transcriptase, and Cas9^20^,^21^,^22^,^23^,^24^,^25^,^26^,^27^. To investigate the importance of these residues, six GmrSD variants D507A, H508A, C517A, N522A, N528A, and N535A were constructed by site-directed mutagenesis and the mutant proteins were purified by nickel column chromatography. Three inactive mutants (D507A, H508A, and N522A) and three partially active variants (C517A, N528A, and N535A) were further purified by heparin column and analyzed by SDS-polyacrylamide gel electrophoresis (SDS-PAGE) (Fig. 4). The protein yield and purity of D507A, H508A, C517A, N528A, and N535A were comparable to the WT enzyme, but the N522A variant showed reduction in protein yield and purity after heparin column. Fig. 4B and 4C shows that GmrSD variants D507A, H508A, and N522A are devoid of endonuclease activity. Variants C517A, N528A, and N535A are partially active (about ~25% to 50% of WT activity. Although the relative activity estimates were crude, C517, N528, and N535 could be ruled out as potential catalytic residues. The relative activity of WT and mutants are summarized in Table 1.

**Figure 5:**
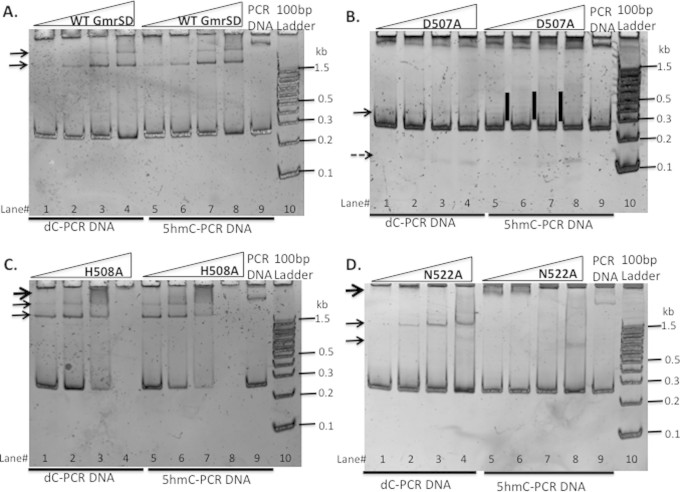
DNA mobility shift assays for WT GmrSD (panel A) and its variants D507A (panel B), H508A (panel C), N522A (panel D) in the presence of 10 mM Mg^2+^ and 1 mM ATP. Bound DNA was resolved in 10% TBE native gels. Two PCR substrates were used: 266-bp dC-DNA (unmodified) and 266-bp 5hmC DNA (modified). Arrows indicate bound complexes (shifted bands).

The mutagenesis results indicate that the critical amino acids of the GmrSD endonuclease catalytic site are likely residues D507, H508, and N522 (See Supplementary Fig. S5 for a model of the predicted active site). This catalytic site is similar to that found in I-HmuI and I-PpoI homing endonucleases and other HNH-family nucleases^19^,^20^,^28^. When a catalytic residue of a REase is mutated, the mutant protein is still capable of DNA binding and this can be detected by DNA mobility shift assay (DNA-REase complexes migrated slower than substrate DNA in native PAGE)^29^,^30^. Purified WT GmrSD, D507A, H508A, and N522A were used to bind a 266-bp PCR fragment containing 5hmC or dC. Two major bound complexes by the WT enzyme were detected and most of the substrate DNA was bound and shifted to the top of gel at high enzyme concentration in an EDTA buffer (no divalent cation, data not shown). It is known that divalent cations can modulate REase specificity: KpnI displays high specificity (low star activity) in Ca^2+^ buffer compared to its specificity in Mg^2+^ and Mn^2+^ buffers^24^. Similarly, divalent cations enhanced the binding specificity of EcoRV catalytic-deficient mutants^31^. Therefore, we examined DNA binding in a buffer with cofactors MgCl_2_ and ATP (binding at room temperature for 10 min to minimize cleavage activity). Fig. 5A shows that two bound complexes were detected on both dC and 5hmC substrates by the WT enzyme. D507A appeared to have reduced DNA binding affinity than the WT enzyme (Fig. 5B) (a large complex is not discernable due to a large DNA fragment present in the substrate DNA). Similar to the WT enzyme, H508A variant also caused gel shift of both 5hmC- and dC-DNAs in the binding assay and appeared to have enhanced binding activity since all the substrate was shifted to the loading well at 60:1 protein to DNA molar ratio (Fig. 5C, lanes 4 and 8). N522A variant protein appeared to have reduced DNA binding affinity to 5hmC DNA: a major bound complex was detected at 100, 250, and 500 ng protein in the gel shift assay for dC-PCR DNA (Fig. 5D, lanes 2–4), but only weak complex formation was detected for 5hmC-PCR DNA at high enzyme concentration (Fig. 5D, lane 8). To further confirm the DNA binding activity of D507A, H508A, and N522A mutant proteins, T4 MluCI (AATT) restriction fragments were used in the DNA mobility shift assay. The WT enzyme and H508A showed similar binding complexes except that at high enzyme concentration all the substrates were shifted up by H508A; D507A and N522A proteins displayed lower affinity and produced shifted/bound complex(s) only at high enzyme concentrations (data not shown). It was concluded that H508A variant is a binding-proficient and cleavage-deficient mutant that fits the definition of catalytic mutant of REases. The binding results on cleavage-deficient mutants D507A and N522A were not conclusive, but suggesting D507 and N522 may be involved in both binding (specificity determination) and catalysis. Further biochemical and structure analysis are needed to refine the roles of D507 and N522 residues.

### Restriction of phage by GmrSD endonuclease

**Figure 6:**
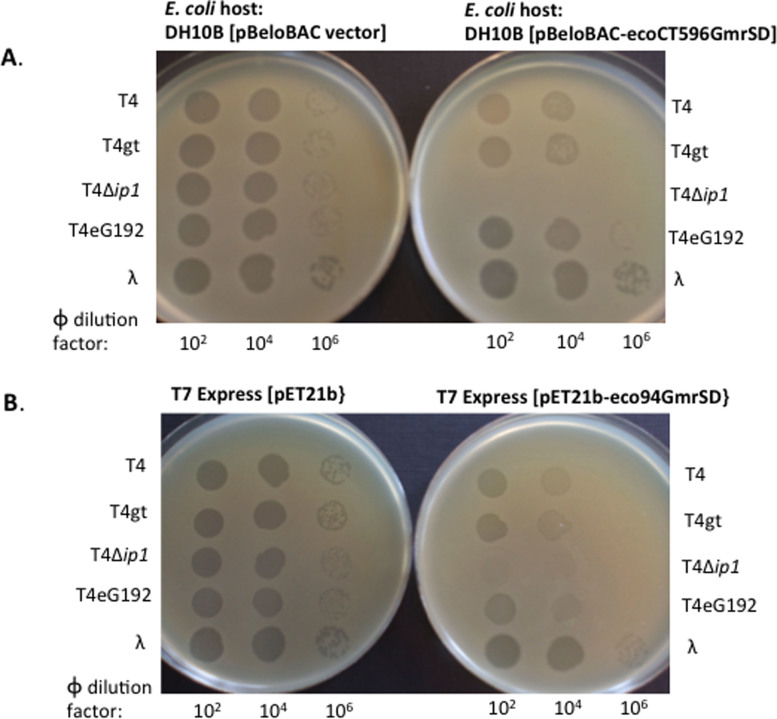
Phage spot tests for T4, T4gt, T4Δ*ip1* (IPI*-deficient), T4 eG192 IPI^+^ (ΔIPII ΔIPIII), and λvir on *E. coli* strains expressing EcoCT596GmrSD or Eco94GmrSD. (A). Two strains DH10B carrying pBeloBAC vector or pBeloBAC-EcoCT596GmrSD were used for comparison. The difference in phage spot (plaques) formation is most evident at 10^6^-fold dilution where EcoCT596GmrSD restricted T4 and T4gt at approximately 5-fold. T4Δ*ip1* (IPI*-deficient) failed to form plaques on EcoCT596GmrSD-expressing strain (input phage ~2–3 × 10^5^ pfu). (B). T7 Express [pET21] and T7 Express [pET21-Eco94GmrSD] strains were used for phage spot tests. Eco94GmrSD moderately restricted T4, T4gt, and T4 eG192, and it did not restrict λvir. T4Δ*ip1* phage was strongly restricted by Eco94GmrSD (no plaque formation at 100-fold dilution, estimated phage input ~2–3 × 10^5^ pfu).

The proposed biological role of GmrSD endonuclease is to serve as a phage exclusion protein (the resident prophage expressing GmrSD to restrict incoming phage with sugar-modified DNA). To counteract GmrSD restriction, T4-like phages evolved inhibitor proteins (internal proteins) such as encapsidated IPI* to inhibit GmrSD activity following T4 DNA ejection into the host cytoplasm^6^,^12^. To determine if Eco94GmrSD was capable of phage restriction, we tested it against WT T4, several T4 mutants and λ phage. We tested its phage plating efficiency using the T7 Express strain containing pET21-*eco94gmrSD* (under constitutive expression, no IPTG added) and used pET21b vector as a control. Consistent with the in vitro result of poor cleavage activity on λ DNA, Eco94GmrSD did not restrict λ phage (Table 2). Eco94GmrSD restricted T4 and T4gt by 15 to 20-fold (Table 2) and this lack of strong restriction could be attributed to the counter measure evolved by T4 phage. T4 phage co-eject inhibitor protein (IPI*, ~360 copies per viral capsid) into the host cytoplasm; this inhibitor protein can antagonize GmrSD nuclease activity and overcome the phage exclusion mechanism, leading to successful phage DNA replication and virus packaging^12^. Consistent with this explanation, Eco94GmrSD strongly restricted T4Δ*ip1* (T4 mutant eG506, IPI*-deficient). T4Δ*ip1* plating efficiency on Eco94GmrSD expressing strain under non-induced condition is in the range of 10^−6^ to 10^−7^. In a control experiment, DH10B cells expressing EcoCT596GmrSD from a single copy pBeloBAC plasmid restricted T4 and T4gt at 5 to 10-fold, and restricted T4 Δ*ip1* phage at ~10^6^-fold. Similarly, the phage restriction activity by phage spot test (10 μl of the diluted phage was spotted on a host cell lawn pre-plated with soft agar) is shown in Fig. 6. Consistent with the phage titers (EOP) in restriction assay, the expression of Eco94GmrSD endonuclease strongly restricted T4Δ*ip1* in the phage spot test (Fig. 6, bottom panel).

### Co-expression of ecoCT596gmrSD and ip1 (IPI*) genes to alleviate toxicity

**Figure 7:**
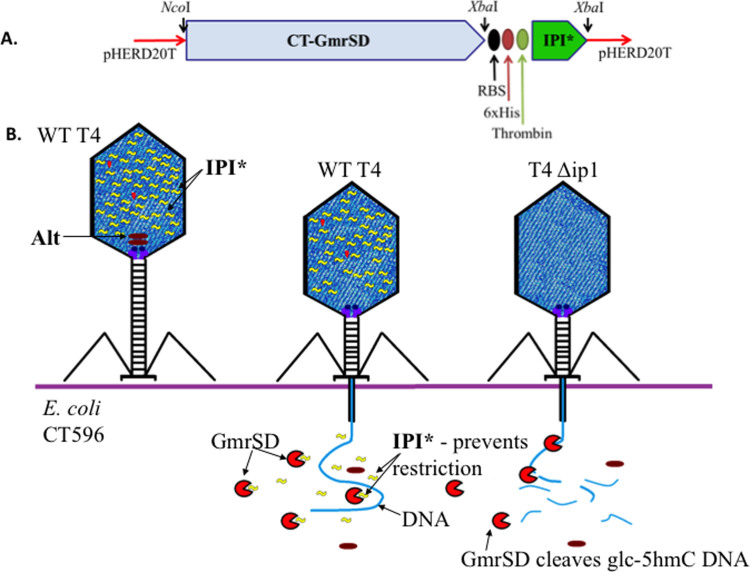
Co-expression of CT596GmrSD and IPI* in the same host and the proposed mechanism of anti-restriction activity of IPI*. (A). Scheme of pHERD20T plasmid construct used to express both CT596GmrSD and T4 IPI* genes and inhibition of GmrSD restriction by IPI* as shown by dual gene expression. The phage restriction activities by GmrSD are summarized in Table 3. (B). A schematic diagram of packaged internal protein IPI* (a.k.a. inhibitor protein) in T4 head and its inhibition of GmrSD restriction activity following DNA/IPI* ejection into host cells.

In the native strain the *ecoCT596gmrSD* expression may be tightly regulated (or because of an unknown detoxification mechanism carried by the surrounding prophage-encoded gene products), *E. coli* CT596 cells show normal growth. In a heterologous host, RecA-deficient *E. coli* DH10B with a single copy plasmid carrying the *ecoCT596gmrSD* gene restricts T4-like glc-5hmC containing phages. However, subcloning of this gene into higher copy plasmids (pBR322-based ColE1 origin) was toxic: successful cloning was apparently enabled by introduction of a stop codon which caused a truncation product GmrS and reinitiation product GmrD with a small deletion between the *gmrS* and *gmrD* genes (Genbank ID: AF493796_1)^6^. (i.e. the two-chain GmrS and GmrD were the result of cloning artifact that still retains endonuclease activity on T4 DNA). Toxicity of the *ecoCT596gmrSD* gene in DH10B was reflected as less than ~10^−6^ survivors by even low level expression from vector pHERD20T; co-expression of the phage IPI* inhibitor protein eliminates this toxicity (see a schematic diagram in Fig. 7A, B), presumably as a result of IPI* neutralizing activity towards GmrSD REase. Table 3 summarizes the growth of different T4-related phages on cell lawns of *E. coli* DH10B expressing either CT596GmrSD or CT596GmrSD plus IPI*. The co-expression of IPI* prevented restriction of phages normally sensitive to EcoCT596GmrSD, e.g., T2. The ultimate purification of the single-chain EcoCT596GmrSD is needed to confirm its nuclease activity and cofactor requirement in vitro. Consistent with the toxicity of over-expressed CT596GmrSD in RecA-deficient *E. coli* cells, constitutive expression of Eco94GmrSD from pBR322 (with a strong ribosome binding site GGAGGT-N6-ATG start codon, under Tc promoter) was quite toxic to RecA minus *E. coli* cells, probably as the result of GmrSD star activity (relaxed nuclease activity on dC and 5mC DNA). The toxicity was reflected by two observations: 100 to 1000 fold-lower transformation efficiency of RecA-deficient cells and poor cell lawn formation during phage infection (data not shown).

## Discussion

### ATP/GTP stimulate endonuclease activity

Although GmrSD endonuclease activity is stimulate by ATP/GTP, there is no predicted ATPase/GTPase domain in the protein by NCBI BlastP analysis. Therefore Eco94GmrSD may carry a novel type of NTPase activity. ATP binding and/or hydrolysis may help with protein translocation, tracking along the DNA substrate, or allosteric activation of the enzyme. It is known that Type IV REase McrBC requires GTP hydrolysis for endonuclease activity, and SauUSI requires ATP hydrolysis for enzyme activity^32^. ATP and GTP also stimulate the endonuclease activity of BceSIV (GCWGC)^33^. More biochemical and structural studies of GmrSD enzyme are necessary to understand the molecular mechanism of NTP/dNTP stimulation of endonuclease activity.

In log phase *E. coli* cells cultured in LB broth the averaged ATP concentration was calculated to be 1.54 mM^16^. GmrSD endonuclease activity is stimulated by a range of ATP concentrations at 0.1–2 mM with the upper limit near the physiological concentration. Conversely, a high concentration of ATP (10 mM) inhibits GmrSD activity by some yet unknown mechanism.

### Eco94GmrSD cut sites

The sequenced cut sites can be summarized as 5hmC N(17–23) G (two 5hmC in the opposite strands separated by 17–23 bp), where cleavage frequently takes place at the semi-symmetric sites 5hmC N(9–11)↓N(8–11)G. Sequencing a large number of cleavage sites (cleavage products) would be required to determine the preferred cut sites. The plasmid-borne modification-dependent REase PvuRts1I prefers to cleave a symmetric site at 5′-5hmC N(11–12)↓N(9–10) G-3′^9^. The crystal structure of PvuRts1I has been solved recently^34^,^35^. Based on the structure, PvuRts1I variants have been engineered to preferentially cleave 5hmC-modified DNA over glc-5hmC DNA^35^. AbaSI endonuclease, a member of the PvuRts1I-family, cleaves DNA containing 5hmC and glc-5hmC, but not DNA containing 5mC or dC. The best substrate for AbaSI cleavage is symmetrically modified 5hmC with a 22-bp spacer (5hmC N22 G), most likely cleaved by a homotetramer^36^.

### Domain organization of Eco94GmrSD

EcoCT596GmrSD and Eco94GmrSD both contain two conserved protein domains DUF262 (Domain of Unknown Function 262) or pfam03235 (Protein family 03235) at the N-terminus, and DUF1524 (pfam07510) at the C-terminus. The DUF1524 family proteins (pfam07510) contain the conserved amino acid motif (D/E/H)HXXP, a motif found in His-metal nuclease superfamily. It is possible that the N-terminal DUF262 domain is involved in DNA recognition and the C-terminal DUF1524 domain is involved in Mg^2+^/Mn^2+^ ion binding and DNA cleavage. A similar domain organization exists in the Type IIS restriction enzymes MnlI and FokI whose N-termini are involved in DNA binding/recognition and C-termini have functions in nuclease catalytic activity^28^,^37^. In contrast, the N-terminus of AbaSI contains a Vsr-like nuclease domain with a single catalytic site and the C-terminal domain harbors the Sra-like 5hmC-binding domain^36^.

### The differences in enzyme properties of the single-chain Eco94GmrSD and two-chain GmrS/GmrD complex

The major differences of the single-chain Eco94GmrSD and the two-chain GmrS/GmrD are: 1) Ca^2+^ is not required for Eco94GmrSD activity, (Ca^2+^ and Mg^2+^ required for the two-chain enzyme), Eco94GmrSD requires Mg^2+^ or Mn^2+^ as a cofactor for catalytic activity; 2) ATP, dATP, and dTTP strongly stimulate the activity of Eco94GmrSD, while UTP, GTP and CTP simulate the activity of the CT enzyme; 3) Eco94GmrSD displays endonuclease activity on 5hmC-modified T4gt or PCR DNA containing 5hmC, but the two-chain GmrS/GmrD has poor activity on 5hmC DNA (4 aa changes and 84-aa deletion may have contributed to this altered specificity); 4) GroEL/ES protein co-purified with Eco94GmrSD similar to the two-chain enzyme, but GroEL/ES proteins can be easily removed by a heparin column chromatography.

### Potential application for Eco94GmrSD endonuclease

GmrSD endonuclease activity may be utilized for in vivo detection of 5mC conversion to 5hmC. For example, *E. coli* *dinD::lacZ* “endo-blue” indicator strain ER1992 is Dcm^+^, McrBC^−^, Mrr^−^, and McrA^−^38 (*dinD*, DNA damage inducible gene D). The *gmrSD* gene could be cloned into pACYC184 plasmid under P_araB_ control (chloramphenicol resistant, Cm^R^). Co-transformation and expression of plasmid (Amp^R^) carrying Tet family dioxygenase will likely covert C5mCWGG to C5hmCWGG in the presence of cofactors^39^. The C5hmCWGG modified sites are substrates for Eco94GmrSD endonuclease. Controlled low expression of GmrSD can cause dsDNA damage and induce host SOS response. The *dinD::lacZ* indicator strain will likely form dark blue colonies on X-gal, Amp, Cm plate. Thus, co-expression of GmrSD and DNA hydroxylase in a *dinD::lacZ* indicator strain could be used to screen functional DNA demethyase variants from cDNA expression library^40^.

### Other *gmrSD* genes associated with Type I and IV restriction systems in the immigration control region (ICR)

**Figure 8:**
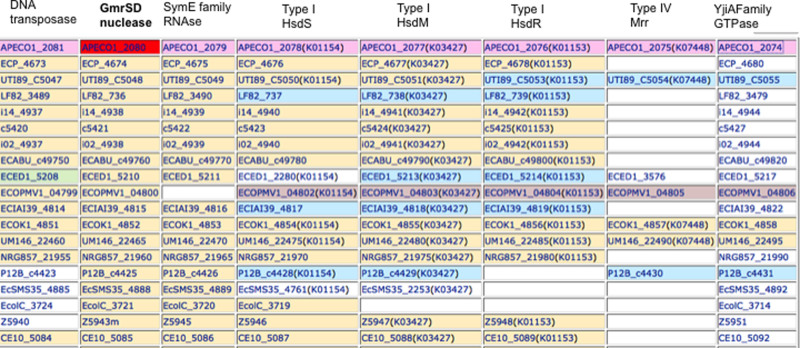
Some putative *E. coli* *gmrSD* genes associated with putative DNA transposases, Type I R-M systems and Type IV restriction systems (Mrr) in bacterial immigration control region (ICR). The “Gene cluster” function on the web server kegg.jp was used to generate the table listing GmrSD homologs and associated DNA transposases and Type I and IV restriction systems. All gene product abbreviations can be found in www.kegg.jp. GmrSD homologs (second column) in some *E. coli* strains: *E. coli* O1 K1 H7 (APEC) = APECO1_2080, *E*. *coli* O6 K15 H31 536 (UPEC) = ECP_4674, *E. coli* O18 K1 H7 UTI89 (UPEC) = UTI89_C5048, *E. coli* LF82 = LF82_736, *E. coli* clone D i14 = i14_4938, *E. coli* O6 K2 H1 CFT073 (UPEC) = c5421, *E. coli* clone D i2 = i02_4938, *E. coli* ABU 83972 = ECABU_c49760, *E. coli* O81 ED1a (commensal) = ECED1_5210, *E. coli* PMV-1 = ECOPMV1_04800, *E. coli* O7 K1 IAI39 (ExPEC) = ECIAI39_4815, *E. coli* IHE3034 = ECOK1_4852, *E. coli* UM146 = UM146_22465, *E. coli* O83 H1 NRG 857C = NRG857_21960, *E. coli* P12b = P12B_c4425, *E. coli* SMS-3-5 (environmental) = EcSMS35_4888, *E. coli* C ATCC 8739 = EcolC_3721, *E. coli* O157 H7 EDL933 (EHEC) = Z5943m, *E. coli* O7 K1 CE10 = CE10_5085. Note, the 5mC (and 5hmC)-dependent type IV restriction genes *mcrB/mcrC* are replaced by *gmrSD* gene in these genomes.

Close homologs to GmrSD are found in some pathogenic *E. coli* strains and more diverged homologs in other bacterial genomes. Fig. 8 shows that in some *E. coli* the GmrSD genes are associated with the immigration control region (ICR) that carries Type I and Type IV Mrr restriction systems^3^,^41^. The Type IV restriction enzyme Mrr restricts methylated DNA with N6mA or 5mC modifications^42^. The 5mC and 5hmC-dependent McrBC endonuclease (*E. coli* K strain) is not present in this locus in these strains. For example, the avian pathogenic *E. coli* strain APEC O1 genome carries two GmrSD homologs, one which is more similar to Eco94GmrSD, the 604- aa APECO1_3911 (93% identity by BlastP) and a more diverged homolog, the 733- aa APECO1_2080 (24% identity by BlastP). Both proteins contain the conserved motifs of DUF262 and DUF1524 characteristic of these enzymes. Like Eco94GmrSD and UTI89GmrSD the APECO1_3911 is likely located on a prophage (its gene is next to the putative phage tail fiber gene APECO1_3910). APECO1_2080, however, is located in an ICR that encodes a putative DNA transposase, endoribonuclease, Type I specificity (hsdS), modification (hsdM), restriction (hsdR), Mrr, and a GTPase. It is possible that APECO1_3911 and APECO1_2080 enzymes are both maintained in the same bacterium to restrict/exclude T-even phages with differences in sugar modifications and/or the two enzymes may display different immunity to the diverse inhibitor proteins (*ip1* locus encoded proteins IPI*) ejected by T4-like phages. Either or both of these functions would provide more fitness to this host than those with only one (or none) GmrSD in resisting phage infection.

## Methods

### Bacterial strains, culture media, cloning vector, and DNA substrates

*E. coli* B strain T7 Express (C2566) (New England Biolabs, NEB) were used for gene cloning and protein expression. *E. coli* cells were grown in LB or phage broth (10 g tryptone, 5 g NaCl, 0.5 g MgCl_2_ in 1 L) supplemented with appropriate antibiotics (Amp at 100 μg/ml, Cm at 33 μg/ml, Km at 50 μg/ml). All restriction and modification enzymes, and DNA polymerases were from NEB. The IMPACT protein expression and purification system (with pTYB1 vector, NEB) was used for GmrSD expression^43^. The *eco94gmrSD* gene (GenBank ID WP_000834395, gene flanked by NdeI and XhoI sites) was synthesized by IDT and inserted in a pIDT (kanamycin resistant, Km^R^) vector. The NdeI-XhoI fragment was sub-cloned into pET21b in fusion with a C-terminal 6xHis tag (N-terminal 6xHis tag not tested) or pTYB1, which allows expression of target protein as a fusion to the intein-CBD tag (in the C-terminus of the target protein). T4, T4gt, and λvir phages were from Lise Raleigh's collection (NEB). T4 eG506 Δ*ip1* (*ip1* gene deletion mutant), and T4 eG192 IPI^+^ (control overlapping ΔIPII ΔIPIII deletion)^44^, IPI deficient *ip1* missense mutations HA35 and KAI^−^12 and *E. coli* strains DH10B containing pBeloBAC vector (Cm^R^) or pBeloBAC-*ecoCT596gmrSD* (a.k.a. DL26)^7^ were from Lindsay W. Black's collection. Cells were grown to mid-log phase in phage broth plus Amp or Cm, concentrated 10-fold and used for phage plating assays or phage spot test. For phage spot tests on *E. coli* lawns, phage stock was diluted by 100-fold serial dilution and 10 μl of the diluted phage was spotted onto the cell lawn.

### Protein purification

For enzyme purification from 2 L of IPTG-induced cells, Eco94GmrSD-6xHis was purified from fast flow nickel-NTA agarose columns (Qiagen). The eluted fractions (5 ml × 6) were analyzed by SDS-PAGE and fractions containing GmrSD were further purified by chromatography through a 5 ml HiTrap heparin HP column (GE Life Sciences). Pooled protein fractions were diluted in a low salt buffer (20 mM Tris-HCl, pH 7.5, 50 mM NaCl, 1 mM DTT, 1 mM EDTA, 20 mM NaCl, 5% glycerol) and loaded onto a heparin HP column using an AKTA FPLC system (GE Life Sciences). Elution was carried out using a salt gradient of elution buffer (50 mM to 1 M NaCl, 20 mM Tris-HCl, pH 7.5, 1 mM DTT, 1 mM EDTA, 5% glycerol). The eluted fractions corresponding to UV absorption peaks were analyzed by SDS-PAGE. Active enzyme fractions were pooled and processed for buffer exchange by running through an Amicon protein concentrator (Millipore). Protein was carefully recovered from the membrane by washing it a few times with a storage buffer (100 mM NaCl, 20 mM Tris-HCl, pH 7.5, 1 mM DTT, 50% glycerol) and the purified enzyme was stored at −20°C.

To determine the optimal temperature for GmrSD-intein-CBD fusion protein production, IPTG-induction (0.5 mM) was carried out at 16°C to 37°C for 4 h to overnight. The protein purification procedure was based on NEB's manual except that DTT-stimulated intein cleavage was carried out at 4°C for 48 h. The target protein was then eluted and analyzed by SDS-PAGE. Eco94GmrSD protein was further purified by chromatography through a heparin column as described above for the 6xHis-tagged version.

### Site-directed mutagenesis of the putative active site residues

Site-directed mutagenesis of *eco94gmrSD* gene was carried out by PCR as described^22^. Mutant alleles were sequenced to confirm the desired mutation(s). Six single or double Eco94GmrSD mutants (in the putative endonuclease catalytic motif PD X_n_ E/D-X-K or PD X_n_ E/D-X-E) located at the N-terminus were constructed this way using pTYB1-*eco94gmrSD*: (1) D217A, (2) E228A/D230A, (3) D249A, (4) E260A/E262A, (5) E271A/E273A, (6) E278A/K280A. Additional six single GmrSD mutants (with C-terminal 6xHis tag) in the putative endonuclease catalytic motif D-H-N located at the C-terminus were also constructed using pET21b-*eco94gmrSD*: (7) D507A, (8) H508A, (9) C517A, (10) N522A, (11) N528A, (12) N535A. Eight mutants were purified by chromatography through nickel-NTA agarose columns. Three inactive mutants (D507A, H508A, N522A), three partially active mutant (C517A, N528A, and N535A), and two double mutants (E271A/E273A, E278A/K280A) were further purified by chromatography through HiTrap heparin HP column.

### DNA binding assay (DNA mobility shift assay)

DNA mobility shift assay was carried out as described^29^. A 266-bp PCR fragment containing 5hmC or dC was used in the binding assays. For binding to glc-5hmC-modified DNA, T4 MluCI restriction fragments (100 to 500 bp mixture) were used in the DNA mobility shift assay. PCR DNA (10 ng) was incubated with 50 ng, 0.1 μg, 0.25 μg, 0.5 μg protein (the molar ratio of GmrSD protein to DNA was estimated at 6.0, 11.9, 29.7, and 59.5, assuming the active form of enzyme is a dimer with DNA) in 1× binding buffer (0.1 M NaCl, 10 mM Tris-HCl, pH 7.5, 1 mM DTT, 0.1 μg of λ carrier DNA) supplemented separately by 1) 5 mM EDTA, 2) 10 mM CaCl_2_, 3) 10 mM MgCl_2_, 4) 10 mM MgCl_2_ and 1 mM ATP, at room temperature for 10 min. Glycerol was added to a final concentration of 10% and the DNA-protein complex was loaded onto a pre-run TBE gel (10%, Life Technologies) and electrophoresis was carried out using 0.5× TBE buffer with gel box emerged in ice water. DNA was stained by SYBR Gold stain (Life Technologies) in 0.5× TBE for 15 min and DNA imaging was carried out on a Typhoon 9400 Imager (GE Life Sciences).

### GmrSD enzyme activity assay

T4 (glc-5hmC), T4gt (5hmC), and λ DNA (Dam^+^ Dcm^+^) or 5hmC-modified PCR DNA were digested with purified GmrSD enzyme in NEB buffer 2 (50 mM NaCl, 10 mM Tris-HCl, 10 mM MgCl2, 1 mM DTT) supplemented with 1 mM ATP at 37°C for 1 h unless specified otherwise. To generate 5hmC-modified PCR DNA substrates (266 bp, 0.5 kb, 1.0 kb, 1.9 kb, 3.8 kb), 5hm-dCTP (Zymo Research) was incorporated into PCR DNA by Taq DNA polymerase during PCR reactions. As a control, similar PCR fragments were also generated using regular dNTP. To test the enzyme requirement for divalent cations, T4 DNA was digested in a basic buffer (50 mM NaCl, 10 mM Tris-HCl, pH 7.5, 1 mM DTT), and supplemented with different metal ions (MgCl_2_, MnCl_2_, CaCl_2_, CoCl_2_, NiSO_4_, ZnSO_4_) as indicated in each digestion. To test NTP stimulation of GmrSD activity, NTP (0.1, 0.5, and 1 mM), dNTP (1 and 10 mM), and γ–S-ATP (1 mM) were added to GmrSD digestions. One GmrSD endonuclease unit is defined as the amount of enzyme required for complete digestion of T4 DNA (170 kb) into fragments less than 500 bp in 1 h at 37°C in buffer 2 supplemented with 1 mM ATP. To examine the optimal temperature for GmrSD activity, T4 DNA was digested at 25°C to 65°C for 30 min in limited digestion.

## Author Contributions

X.H. performed initial experiments on Eco94GmrSD enzyme purification using the IMPACT system, enzyme activity assays, and substrate preference. V.H. purified WT and mutant enzymes and performed site-directed mutagenesis of the putative catalytic site, mutant activity assay, and DNA binding assays on modified substrates. J.T. and L.W.B. contributed the work on expression, purification, and activity assays for UTI89_C2960 protein, cloning of the single chain EcoCT596GmrSD gene with or without IPI* gene and prophage analyses. F.X. produced PCR DNAs containing 5hmC or dC. S.-Y.X. performed GmrSD activity assays in the presence of various divalent cations/NTP/dNTP/temperatures, phage-plating assay, and phage spot test. Y.G. constructed the model of GmrSD catalytic site. S.G. purified the WT GmrSD protein for trial crystallography. X.H., V.H., J.A.T. and S.-Y.X. analyzed data. S.-Y.X. and L.W.B. wrote the manuscript.

## Additional Information

**How to cite this article**: He, X. *et al.* Expression and purification of a single-chain Type IV restriction enzyme Eco94GmrSD and determination of its substrate preference. *Sci. Rep.* **5**, 9747; doi: 10.1038/srep09747 (2015).

## Supplementary Material

Supplementary Information Supplement information

## Figures and Tables

**Table 1:** Summary of endonuclease activity on T4 DNA and protein expression levels of WT and mutant forms of Eco94GmrSD

Enzyme	Endonuclease activity	Protein expression level
WT	+++ Active (100%)	++
**C-terminus mutants (1 to 6)**		
1) D507A	**Inactive** (binding+/−)	++
2) H508A	**Inactive** (binding+)	++
3) C517A	+ Partially active	++
4) N522A	**Inactive** (binding+/−)	+
5) N528A	+ Partially active	++
6) N535A	+ Partially active	++
Possible Eco94GmrSD endonuclease catalytic site: D507-H508-N522. Binding+, bound/shifted complexes detected at all four protein concentrations tested; binding+/−, bound/shifted complexes were detected only at high enzyme concentrations.		

**Table 2:** Restriction of phages by Eco94GmrSD endonuclease

	PFU/ml on T7 Express [pET21]^a^	PFU/ml on T7 Express [pET21-gmrSD]^a^	How many fold of restriction by GmrSD^b^
T4	4.4 × 10^9^ (±0.3 × 10^9^)	3.0 × 10^8^ (±0.2 × 10^8^)	15-fold
T4gt	4.2 × 10^9^ (±0.3 × 10^9^)	2.1 × 10^8^ (±0.2 × 10^8^)	20-fold
T4 Δ*ip1* (IPI*-deficient)	2.2 × 10^9^ (±0.2 × 10^9^)	Less than 10^3^ (No plaque at 100-fold dilution)	More than 10^6^-fold
λvir	2.8 × 10^9^ (±0.4 × 10^9^)	3.0 × 10^9^ (±0.4 × 10^9^)	No restriction
T4 eG192 (IPII^−^ IPIII^−^)	5.8 × 10^9^ (±0.2 × 10^9^)	2.7 × 10^8^ (±0.5 × 10^8^)	21-fold
^a.^PFU/ml are average of three plating numbers (SD± is shown in parenthesis).			
^b.^Ratio of PFU/ml on vector strain and Eco94GmrSD expressing host.			

**Table 3:** Growth of different T4-related phages on cell lawns of *E. coli* DH10B expressing either CT596GmrSD or CT596GmrSD and IPI*

Phage	Phage has IPI*	pHERD20T^a^	pHERD20T + CT596GmrSD	pHERD20T + CT596GmrSD + IPI* (see Fig. 7)
T4	Yes	+	+	+
T4*ip1*HA35	No	+	−	+
T4eG192	Yes	+	+	+
T4eG506	No	+	−	+
RB15	No	+	+	+
T2^b^	No	+	−	+
RB49 (no 5hmC)	No	+	+	+
T4*ip1*KAI^−^	No	+	−	+
^a.^A control of host (DH10B) containing the expression vector alone. Cultures were induced with 0.4% arabinose. A series of spots containing 10^2^, 10^4^, 10^5^ and 10^8^ phage particles were examined for growth after overnight incubation. −, indicates no phage growth; +, indicates EOP (efficiency of phage plating) >0.6; +/− indicates EOP < 0.6.				
^b.^The co-expression of IPI* prevented restriction of phages normally sensitive to GmrSD, e.g., T2.				
