# Supplementary material for: Expression and purification of a single-chain Type IV restriction enzyme Eco94GmrSD and determination of its substrate preference
Source: Sci Rep. 2015 May 19;5:9747. doi: 10.1038/srep09747 (PMC4437046; doi:10.1038/srep09747)
Supplement: Supplementary Information — Supplement information [file srep09747-s1.doc]

**Supplementary information (7 figures and 2 tables)**

**Expression and purification of a single-chain Type IV restriction enzyme Eco94GmrSD and determination of its substrate preference**

Xinyi He^1, 2^#, Victoria Hull^1^#, Julie A. Thomas3^, Xiaoqing Fu^1^, Sonal Gidwani^1^, Yogesh K. Gupta^4^, Lindsay W. Black^3^, and Shuang-yong Xu^1^*

1.      New England Biolabs, Inc., 240 County Road, Ipswich, MA 01938, USA.

2.      State Key Laboratory of Microbial Metabolism, and School of Life Sciences & Biotechnology Shanghai Jiao Tong University, 1954 Huashan Road, Shanghai, 200030, China

3.      Department of Biochemistry and Molecular Biology, University of Maryland School of Medicine, 108 North Green St, Baltimore, MD 21201-1503, USA.

4.      Department of Structural and Chemical Biology, Icahn School of Medicine at Mount Sinai, Box 1677, 1425 Madison Avenue, New York, NY 10029, USA.

#These authors contributed equally to this work.

^ Current address:

College of Science, Thomas H. Gosnell School of Life Sciences, Rochester Institute of Technology, 85 Lomb Memorial Drive, Rochester, NY 14623-5603.

*Corresponding author

Dr. Shuang-yong Xu

Telephone: 1-978-380-7287

Fax: 1-978-921-1350

Email: [xus@neb.com](mailto:xus@neb.com)

**Temperature profile of Eco94GmrSD and buffer preference**

Eco94GmrSD partial digestion of T4 DNA was carried out at 25°C, 30°C, 37°C, 42°C, 50°C, and 65°C for 30 min and the digested DNA was analyzed by agarose gel electrophoresis. The enzyme displays highest activity at 37°C (major cleavage products close to 500 bp); limited digestion occurred at 25°C, 30°C, and 42°C. The enzyme was inactive at 50°C and 65°C (**Supplementary Fig. S3**). A low residual activity was detected at 50°C in 1 h digestion (data not shown). The Eco94GmrSD enzyme was active in NEB buffers 2 and 4, but only partially active in NEB buffer 1 (no salt buffer) and buffer 3 (high salt buffer) with less than 20% activity compare to that in buffer 2.

**Purification of Eco94GmrSD from chitin columns**

We purified fusion protein Eco94GmrSD-intein-CBD from cells in co-overexpression of GroEL/GroES. IPTG and L-Arabinose induced cell lysates were loaded into a chitin column and washed extensively with the column buffer. Following DTT-catalyzed intein cleavage of GmrSD from intein-CBD tag, the target protein was eluted (note: inclusion of 1 mM ATP in the chitin column elution buffer appeared to increase the amount of eluted GmrSD protein), combined and diluted into 50 mM salt and further purified through heparin column chromatography. The final purified enzyme yield was estimated at 0.8 mg/L of IPTG-induced cells, about 5-fold lower than 6xHis tagged version. Similar to the 6xHis-tagged single-chain enzyme, the chitin column purified enzyme showed low activity on T4 and T4gt DNA in the absence of ATP and no activity on λ DNA. In the presence of ATP (1 mM), however, the endonuclease activity was strongly stimulated on T4 and T4gt DNAs (data not shown). The chitin-column purified Eco94GmrSD enzyme is also active in cleaving PCR DNA substrates with 5hmC or glc-5hmC and inactive in cleaving unmodified PCR DNA (data not shown). The chitin/heparin column purified enzyme displayed a slightly lower specific activity (~200 U/mg protein). One GmrSD endonuclease unit is defined as the amount of enzyme required for complete digestion of T4 DNA (170 kb) into fragments less than 500 bp in 1 h at 37°C in buffer 2 supplemented with 1 mM ATP.

**GmrSD cleavage site determination**

We cloned and sequenced DNA fragments from Eco94GmrSD-digested T4gt DNA. The common feature of these cut sites appeared to be 5hmC N(17-23) G where two 5hmC are present on opposite strands separated by 17-23 bp (see **Supplementary Table S1**) and cleavage takes place mostly at the symmetric sites 5hmC N(9-11)↓N(9-11)G. We also tested digestion of smaller PCR products (60 bp) that contained 0 to 9 modified 5hmC despite prior knowledge of low activity on short 5hmC-modified substrates. Only one PCR product (PCR#1: 5hmC N20 G) was partially digested into smaller products (30-32 bp); other PCR substrates with 0-4 modified 5hmC (PCR#2-#8) were not digested at prolonged digestion (4 h at 37^o^C, data not shown) (**Supplementary Fig. S4**). Sequencing more cleavage products is necessary to pinpoint the precise recognition sequence, sequence context effect on cleavage efficiency (if any), predominant cut sites, and the optimal spacer for Eco94GmrSD endonuclease.

**Computer-generated homology model of active site residues in Eco94GmrSD**

In order to visualize the probable location of these predicted catalytic residues (D507/H508/N522) in 3D space, we calculated a theoretical model of the C-terminal fragment (residues Val435-Asp629) of Eco94GmrSD (**Supplementary Fig. S5**) using the structure of HNH homing endonuclease I-HmuI (pdb id 1U3E) as a template using program MODELLER ^1^. As shown in **Supplementary Fig. S5**, Asn522 and Asn528 reside in the loop regions flanking the Asp507/His508 dyad located in the central helix. DNA binding to GmrSD may likely induce conformational changes in the loop regions surrounding the central helix and therefore, either enhances the proximity of these two asparagine residues towards Asp507/His508 for their active role in catalysis or it enhances DNA/metal ion binding ^2^. The N-terminal residues E271, E273, E278, and K280 are not modeled in here.

**Site-directed mutagenesis of putative catalytic residues at the N-terminus**

There are two commonly found catalytic site motifs in restriction enzymes; i.e. PDX(10-24)D/EXK (X= any amino acid residues, in BamHI and some REases the catalytic motif is PDX(10-24)EXE); The other well-known nuclease catalytic site is H-N-H (H-N-K or H-N-N) ^3-5^. By amino acid sequence inspection we identified several catalytic motif candidates, such as D217-X(10)-E228-X-D230 (E228 and D230 residues are also located in the sequence LEAE228DD330AQ motif similar to the LAGLIDADG catalytic site found in some homing endonucleases) ^6^ and D249-X(10)-E260-X-E262, D249-X(21)-E271-X-E273, or E-X(4-17)-E278-X-K280 conforming to PDX(10-24)D/EXK and PDX(10-24) EXE motifs. To find out the importance of these residues, we constructed six mutants by site-directed mutagenesis: (1) D217A, (2) E228A/D230A, (3) D249A, (4) E260A/E262A, (5) E271A/E273A, (6) E278A/K280A. The mutant enzymes were purified by chitin column chromatography (from 2 L of IPTG-induced cells at 18^o^C, with co-expression of GroES/GroEL protein from pGro7). The purified enzymes were used to digest T4 DNA. The enzyme variants D217A, E228A/D230A, D249A were active in cleaving T4 DNA (data not shown), indicating that these four residues are not critical for enzyme activity. GmrSD variants E260A/E262A, E271A/E273A, and E278A/K280A were also purified. Number 4 variant E260A/E262A showed partial endonuclease activity. Mutant enzymes E271A/E273A and E278A/K280A show no detectable endonuclease activity on T4 DNA (data not shown). Later on, the NdeI-XhoI fragments encoding the double mutants E271A/E273A, E278A/K280A were cloned into pET21b and the double mutant enzymes were purified by chromatography through nickel and heparin columns. The 6xHis-tagged E271A/E273A was inactive in cleavage assay, a similar result obtained with chitin column-purified protein. The 6xHis tagged purified E278A/K280A, however, showed a low but detectable activity (slight smearing at high enzyme concentration, see **Fig. 4**, panel C). The two conflicting results of intein-CBD-tagged protein (cleaved off later by DTT) and 6xHis-tagged E278A/K280A may be reconciled by the observation that the WT GmrSD purified by the two methods varies in specific activity by about 2-fold (probably as a result of mis-folding of the fusion protein GmrSD-intein-CBD). The six GmrSD mutant activities are summarized in **Supplementary Table S2**. The mutagenesis data suggested that the GmrSD catalytic sites are not likely to be located in residues D217-X(10)-E228-X-D230 or D249-X(10)-E260-X-E262 even though this conserved motif is found in some REases ^7,8^.

We performed DNA mobility shift assay for the two double mutant proteins. **Supplementary Fig. S6** shows that both mutants were still capable of DNA binding. 3-4 shifted bands (bound complexes) were detected for E271A/E273A protein and 2-3 bound complexes were observed for E278A/K280A protein. This result was quite unexpected. Is it possible that GmrSD carries two catalytic sites, one located at the N-terminus domain involving E271, E273, E278, and K280 residues, and a second catalytic center located at the C-terminus (D507, H508, N522), or just one composite catalytic site involving amino acids residues of both N-terminal and C-terminal domains? It was noted before that the two-chain GmrS/GmrD enzyme requires both subunits for endonuclease activity. Neither one subunit alone displays nuclease activity ^9^. More genetic, biochemical, and structural studies are necessary to pinpoint the exact contribution of aa residues E271, E273, E278, and K280 to DNA binding and/or catalysis. Secondary structure prediction by the Phyre server indicated that this N-terminal region shows weak similarity to the “winged helix” DNA-binding domain found in transcription regulators (repressors) and TnsA (transposase) (data not shown).

**Expression and purification of single-chain GmrSD from *E. coli* UTI89 strain**

The GmrSD homolog from *E. coli* UTI89 (ORF UTI89_C2960) was cloned into the IMPACT vector pTYB2 (NEB). The construct was transformed into BL21(DE3) and the insert confirmed by DNA sequencing. For protein purification cells were propagated in 0.5 L of solubility media (6) to OD_600_ 0.5 and then induced with 1 mM IPTG overnight at 18°C. Cells were pelleted by centrifugation (10,000 g, 8 min, 4°C), and resuspended in 15 ml of column buffer buffer (20 mM Tris-HCl, pH 8.0, 0.5 M NaCl, 1 mM MgCl_2_). Cells were lysed via sonication, and cell debris removed by centrifugation. The supernatant was loaded onto a chitin bead column (NEB) and washed with column buffer. Purified protein was cleaved from the chitin-binding domain (CBD) in the presence of 50 mM DTT, eluted from the column, and dialysed against dialysis buffer (20 mM Tris-HCl, pH 8.0, 200 mM NaCl, 5 mM MgCl_2_). The protein was further purified by chromatography through a DEAE column to remove the contaminating protein GroEL.

As with the EcoCT596GmrSD enzymes, the UTI89_C2960 protein co-purified with GroEL (identified by mass spectrometry and gel migration, data not shown). The contaminating GroEL was removed via DEAE column purification (**Supplementary Fig. S7**). CD and light scattering Mol. Wt. analyses of this protein at NIH (Paul Wingfield and Ira Palmer, unpublished result) showed the protein to be monomeric (in the absence of substrate DNA), compact, folded and with high α-helical content consistent with its predicted secondary structure.

The purified UTI89_C2960 protein was inactive on phage DNA substrates including T4, T2 and T6. No cleavage activity, or evidence of binding was observed with synthetic 20-mer substrates of 5mC and 5hmC, the latter with and without β-glucosylation (provided by John Dunn) (data not shown). No activity was observed despite various assay conditions, including different times, temperatures (room temperature to 37°C), different reaction buffers (including all available NEB buffers, the two-chain GmrSD nuclease buffer) and different supplements, including NTPs (GTP, UTP, ATP), SAM, BSA or metal ions (data not shown).

As with some GmrSD homologs the C2960 gene is encoded on a Phast (Phage search tool: http://phast.wishartlab.com) diagnosed *Shigella* phage SfII-like prophage region (52.4 kb) of the UTI89 genome (GenBank ID: CP000243.1, co-ordinates 2888714-2941121). Although this UTI89 prophage appears to be intact, including having all the structural elements (tail genes, including a sheath protein, capsid genes and a terminase) typical of a myovirus, we were unable to detect this prophage after induction attempts using UV and mitomycin C by plating on UTI89 and CT596 indicator strains. A second GmrSD homolog (UTI89_C5048) in the same strain has not been characterized. In phage restriction assays, *E. coli* UTI89 strain is incapable of restricting T4 or T4 IPI^-^ phages but restricts some other T4-even phages, apparently due to different enzyme specificity and immunity to IPI* inhibition ^10^.

**Figure S1.**  ClustalW2 multiple sequence alignment of three GmrSD homologs.

The two-chain GmrS/GmrD was first discovered in *E. coli* CT596. Eco94GmrSD is from *E.* *coli* STEC_94C and shares 99% sequence identity to the CT enzyme. Eco89GmrSD is encoded by the uropathogenic *E. coli* UTI89 (UT enzyme). “* or :” indicate identical or similar aa residues. “xxxxx” indicates the missing 84-aa residues (connector of GmrS and GmrD) in the two-chain CT enzyme. The important residues ExEx(4)ExK and putative catalytic residues D-H-N are shown in bold (red).

GmrS (DUF262)

GmrD (DUF1524)

GmrSD (DUF262)

GmrSD (DUF1524)

(connector)

EcoCT596

GmrS/GmrD

Eco94

GmrSD

DH-N motif

CT596GmrS/GmrD MKSETLTIQQIFQNQRQYRVPFYQRAYVWTQRNQWSALLEDIFEKAQSRLSGTKPTPHFL 60

CT596GmrSD MKSETLTIQQIFQNQRQYRVPFYQRAYVWTQRNQWSALLEDIFEKAQSRLSGTKPTPHFL 60

Eco94GmrSD MKSETLTIQQIFQNQRQYRVPFYQRAYVWTQRNQWSALLEDIFEKAQSRLSGTKPTPHFL 60

UTI89GmrSD MKSETLTVQQLFQDRRQYCVPFYQRAYVWTQQDQWSALLEDILEKVQSRLSGTKPTPHFL 60

*******:**:**::*** ************::*********:**.**************

CT596GmrS/GmrD GAVVLEPQLKNSLLGVDTIHIIDGQQRLTTLQYILA**SLA**SIRLSLRATGLSELEGLVLTC 120

CT596GmrSD GAVVLEPQLKNSLLGVDTIHIIDGQQRLTTLQYILA---SIRLSLRATGLSELEGLVLTC 117

Eco94GmrSD GAVVLEPQLKNSLLGVDTIHIIDGQQRLTTLQYILA---SIRLSLRATGLSELEGLVLTC 117

UTI89GmrSD GAVVLEPQSKKGLLGVDSIHIIDGQQRLTTLQYVLA---SIRLALRATDLSSLEALISPC 117

******** *:.*****:***************:** ****:****.**.**.*: .*

CT596GmrS/GmrD LKNTNEATMRNKKVECFKLWPTFRDQTHFIQSLNVDNIDDLRNVFSDSFTQHGTLRKHFN 180

CT596GmrSD LKNTNEATMRNKKVECFKLWPTFRDQTHFIQSLNVDNIDDLRNVFSDSFTQHGTLRKHFN 177

Eco94GmrSD LKNTNEATMRNKKVECFKLWPTFRDQTHFIQSLNVDNIDDLRNVFSDSFTQHGTLRKHFN 177

UTI89GmrSD LKNSNEDTMRNKEVERFKLWPTFRDQTHFIQSFNVENIDDLRDVFSDSFTQHGTLRKHFN 177

***:** *****:** ****************:**:******:*****************

CT596GmrS/GmrD HPPSLEALWFFTEAFIKWIKIENHSPQENAVALIEAVLTDLKLVSIFLEAEDDAQIIFET 240

CT596GmrSD HPPSLEALWFFTEAFIKWIKIENHSPQENAVALIEAVLTDLKLVSIFLEAEDDAQIIFET 237

Eco94GmrSD HPPSLEALWFFTEAFIKWIKIENHSPQENAVALIEAVLTDLKLVSIFLEAEDDAQIIFET 237

UTI89GmrSD HPPSLEALCFFTEAFIKWIKIENHSPQENAVALIEAVLTDLKLVSIFLEAEDDAQIIFET 237

******** ***************************************************

CT596GmrS/GmrD LNGRGAELHATDLIRNYIFMCAEHENINAIELYENEWKIFEDKYWSEKQRRGRINKPRME 300

CT596GmrSD LNGRGAELHATDLIRNYIFMCAEHENINAIELYENEWKIFEDKYWSEKQRRGRINKPRME 297

Eco94GmrSD LNGRGAELHATDLIRNYIFMCAEHENINAIELY**E**N**E**WKIF**E**D**K**YWSEKQRRGRINKPRME 297

UTI89GmrSD LNGRGAELHATDLIRNYIFMCAEHENINAIELYENEWKSFEDKYWSEKQRRGRINKPRME 297

************************************** *********************

CT596GmrS/GmrD WLVHATLQSERQ**Q**xxxxxxxxxxxxxxxxxxxxxxxxxxxxxxxxxxxxxxxxxxxxxxx 313

CT596GmrSD WLVHATLQSERQREIDLSRLYNEYRDYVSKDLPSQRADLQVKRLKQYASQYKELVGGFGT 357

Eco94GmrSD WLVHATLQSERQREIDLSHLYNEYRDYVSKDLPSQRADLQVKRLKQYASQYKELVGGFGT 357

UTI89GmrSD WLVHATLQSERQREIDLSRLYNEYRDYVSKDLSSQRADLQVKRLKQYASQYKELVDGFGT 357

************:

CT596GmrS/GmrD xxxxxxxxxxxxxxxxxxxxxxxxxxxxxxxxxxxxxMYNDLVSYVVRRSVCGLTPKNYN 336

CT596GmrSD TPISHFGHRIAAYDVTTLYPLALFISIANIADDEKAAMYNDLVSYVVRRSVCGLTPKNYN 417

Eco94GmrSD TPISHFGHRIAAYDVTTLYPLALFISIANIADDEKAAMYNDLVSYVVRRSVCGLTPKNYN 417

UTI89GmrSD TPISHFGYRIADYDVTTLYPLALFISIANIADDEKAAMYNDLVSYVVRRAVCGLTPKNYN 417

************:**********

CT596GmrS/GmrD NVFMNVLRHLSKTEISSVELRNILNSLNGEASRWPGDSEFLNACINAPLYPGRLDAPKMR 396

CT596GmrSD NVFMNVLRHLSKTEISSVELRNILNSLNGEASRWPGDSEFLNACINAPLYPGRLDAPKMR 477

Eco94GmrSD NVFMNVLRHLSKTEISSVELRNILNSLNGEASRWPGDSEFLNACINAPLYPGRLDAPKMR 477

UTI89GmrSD NVFMNVLRHLAKTEISSVELRNILNNLNGEASRWPGDSEFLNACINAPLYPGRLDAPKMR 477

**********:**************.**********************************

CT596GmrS/GmrD SMLTELERELCRQVKTEKPDVPNLSNLDIDHLMPQSWYSCWPLENGHMVTNSDATVMNQI 456

CT596GmrSD SMLTELERELCRQVKTEKPDVPNLSNLDIDHLMPQSWYSCWPLENGHMVTNSDATVMNQI 537

Eco94GmrSD SMLTELERELCRQVKTEKPDVPNLSNLDI***DH***LMPQSWYSCWPLE***N***GHMVTNSDATVMNQI 537

UTI89GmrSD SMLTELERELCRQVKTEKPDVPNLSNLDIDHLMPQSWYSCWPLENGRMVTNSDATVLNQI 537

**********************************************:*********:***

CT596GmrS/GmrD VLSGTDLTPEQLLVRKRQQAIATLGNLTLLNLSVNRSVQNAVFLKKRDALIVHTNLRLNI 516

CT596GmrSD VLSGTDLTPEQLLVRKRQQAIATLGNLTLLNLSVNRSVQNAVFLKKRDALIVHTNLRLNI 597

Eco94GmrSD VLSGTDLTPEQLLVRKRQQAIATLGNLTLLNLSVNRSVQNAVFLKKRDALIVHTNLRLNI 597

UTI89GmrSD VLSGTDLTPEQLLVRKRQQAISTLGNLTLLNLSVNRSVQNAVFLKKRDALIVHTNLRLNI 597

*********************:**************************************

CT596GmrS/GmrD PLILKDKWDESEIQERGKKLGEIALKVWPKYD 548

CT596GmrSD PLILKDKWDESEIQERGKKLGEIALKVWPKYD 629

Eco94GmrSD PLILKDKWDESEILERGKKLGEIALKVWPKYD 629

UTI89GmrSD PLIVKDKWDEDEILERGKKLGEIALKVWPKHD 629

***:******.** ****************:*

**Fig. S2.** GmrSD digestion of T4 and λ DNAs in MnCl_2_ buffer (50 mM NaCl, 10 mM Tris-HCl, pH 7.5, 1 mM DTT, 0.1 to 1 mM MnCl_2_) with or without ATP (1 mM).

1. GmrSD digestion of T4 DNA in MnCl_2_ buffer (lanes 1-8), and in 10 mM MgCl_2_ buffers: NEB buffer 2 and 4 (lanes 9-10).
2. GmrSD digestion of λ DNA (Dam^+^ Dcm^+^) in MnCl_2_ buffer (lanes 1-8), and in NEB buffer 2 and 4 (lanes 9-10). Although λ DNA was modified by the Dcm methylase (C^5m^CWGG), it does not contain modified bases 5hmC or glc-5hmC. Thus, the partial digestion of λ DNA in MnCl_2_ buffer (random fragment smearing) by GmrSD could be interpreted as the result of relaxed specificity or star activity.

**Figure S3.** Temperature preference for GmrSD digestion.

T4 DNA (1 μg) was partially digested by GmrSD (2 μg) in NEB buffer 2 at 25°C, 30°C, 37°C, 42°C, 50°C, and 65°C for 30 min with 1 mM ATP. Some DNA was trapped in the loading well due to heat-denatured protein at 50°C and 65°C. DNA products were resolved on a 1% agarose gel.


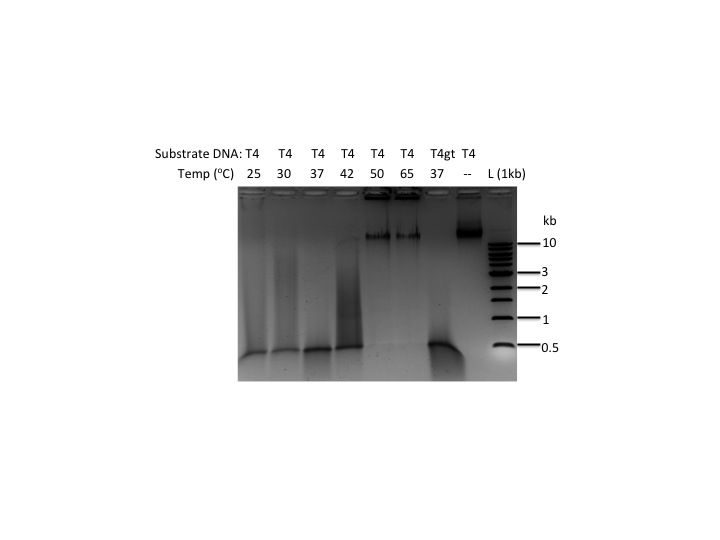


**Figure S4**. Eco94GmrSD digestion of PCR products containing 5hmC. The middle portion of the duplex oligos (#1-#7, 22 bp boxed region) contains 5hmC incorporated during PCR amplification. The forward and reverse primers are underlined. Only #1 PCR substrate (5hmC N20 G) was partially digested by Eco94GmrSD (chitin column purified). #2, #3, and #4 substrates [5hmC N(9-10) G] and other duplex oligos were not digested. S=substrate; P=cleavage product.


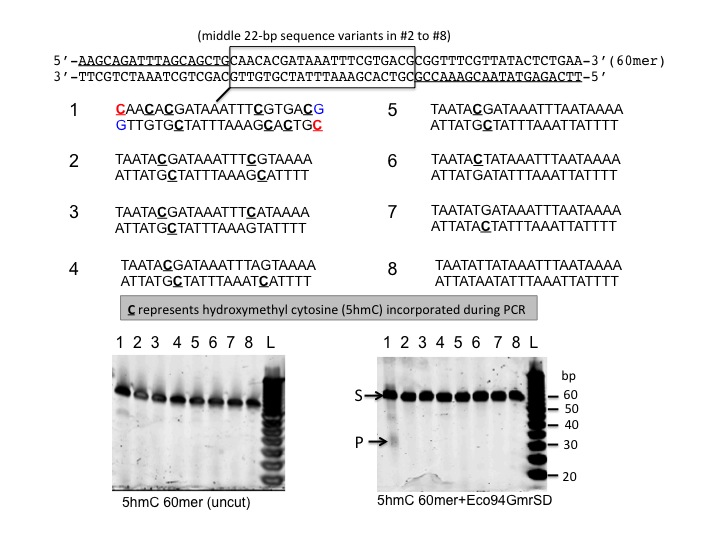


**Figure S5**. A cartoon illustration of a homology-based model of the putative catalytic domain (amino acid residues Val435-Pro546) of Eco94GmrSD. The predicted catalytic residues (D507/H508/N522) are shown as blue sticks. The side chains of two other residues D505 and N528 near the predicted active site are also shown.


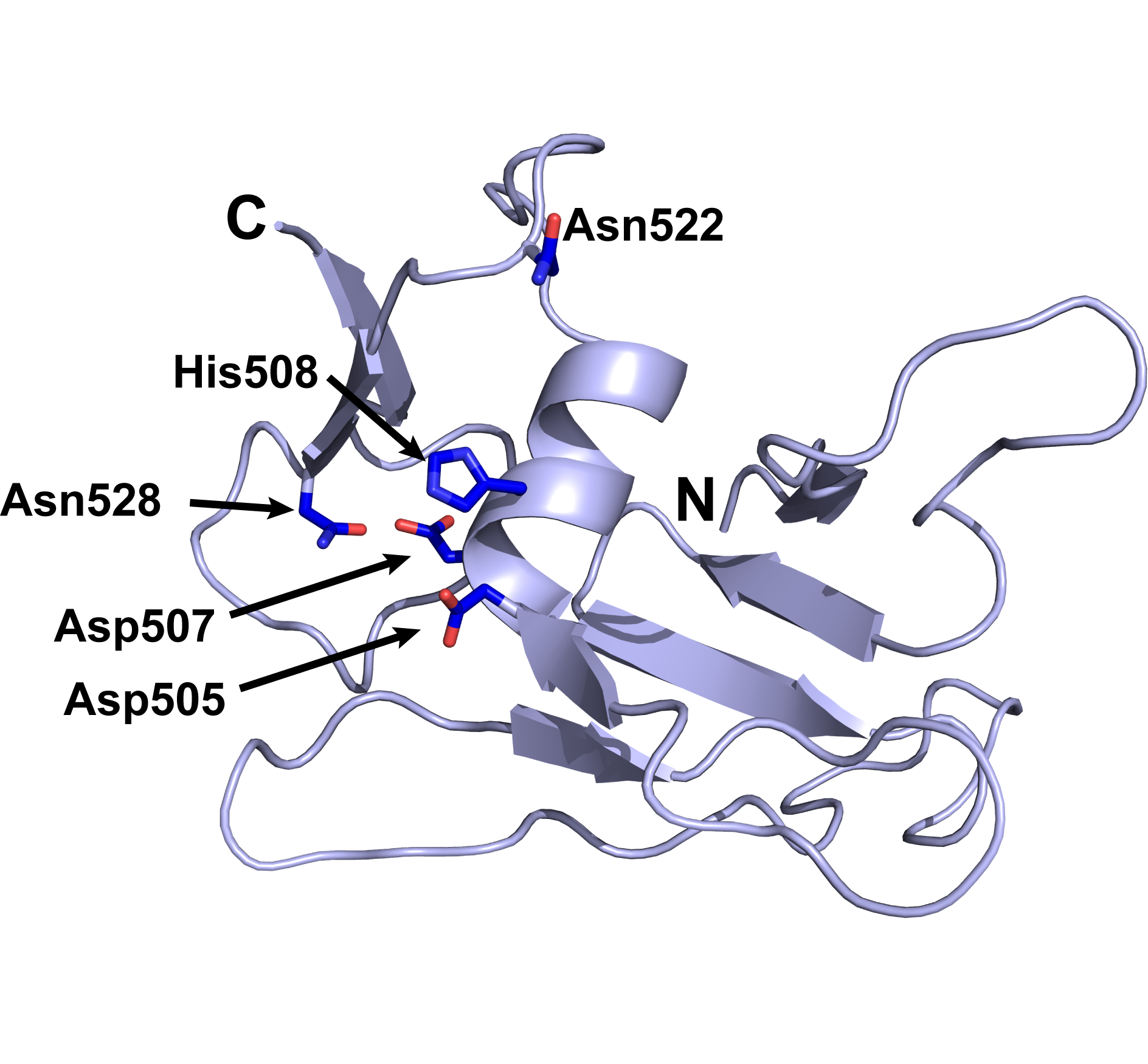


**Fig. S6.** DNA mobility shift assay for GmrSD variants E271A/E273A (panel A), E278A/K280A (panel B), C517A (panel C), N528A (panel D), N535A (panel E). Arrows indicate shifted bands (bound complexes). Four concentrations of protein (0.1, 0.25, 0.5, 1 μg) were used to bind 10 ng of unmodified (dC) or modified (5hmC) DNA substrates.

**Figure S7.** SDS-PAGE analysis of purified EcoUTI89GmrSD after chromatography through (A) a chitin column, and followed by then (B) a DEAE column. Black arrow indicates the purified single-chain UT enzyme (~72 kDa). White arrow in (A) indicates co-purifying GroEL. The endonuclease activity (negative) is not shown here.


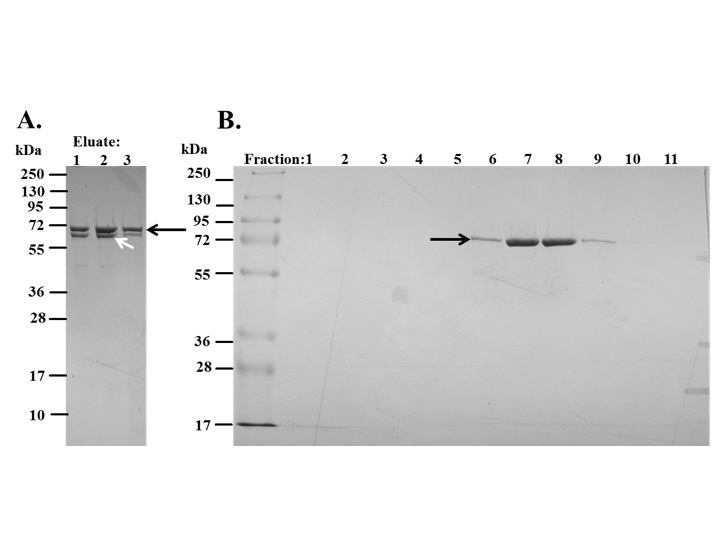


**Supplementary Table S1. DNA sequence of T4gt insert (underlined) after GmrSD digestion and cloning in pUC19. Cut sites were inferred from the cloning junctions.**

_______________________________________________________________________________

**Cleavage sites inferred from cloned DNA junctions^a^ Cut site^b^** _______________________________________________________________________________

CATTAGCTCAATTCCGTCAAGAACA↓TACTGCAGCGTTTGAAGGGACTTCT 5hmC N10↓N9 G

CTAATCCATCAAAATCCAGTTTAAG↓ATGCGATATTTGATCGCCATCACCT 5hmC N9↓N11 G

ATTCAGAGGTAATTTCTTCTGAATA↓TGCTGTCATGCGAGTTGGGAATGAA 5hmC N9↓N9 G

ATTTTCTACTGATTCAGTATTCCAC↓AAACACCAATACGTAATTGGCTTAT 5hmC N10↓N13 G

ACTCGGGGGAAAATCTCCCGAGTAA↓TCAATATCGTTGGATTAATTTAGGA 5hmC N10↓N11 G

TTTAATAATCTTTTTCATTTTCTGT↓TCCTCCATAGTTGATAGGGTAATAG 5hmC N9↓N9 G

TCATATTGCCCATCAATTTCAACGA↓CTTCACCACGAAATTCTGGGAATTC 5hmC N11↓N9 G

TTTTACCCAGCTTACCAGTTATATG↓GGAATGATGCAAGATATGCTCTCTC 5hmC N10↓N8 G

AACACTATTTCTCATTTAATTCTAC↓CTGACATCGTTATGCGTTATTTAGT 5hmC N12↓N8 G

GATTTAGCAGTTTCAACACGATAAA↓TTTCGTGACGAAGTTTAGTTATACT 5hmC N11↓N9 G

ATTACAAAGCAATCCACGCACCTTT↓CCTGCTTTTGGTCCATTTAATTCAT 5hmC N10↓N9 G

GTTCGTATAGTATCAACAGACCGAG↓ATTCTAAAGGAATTGGTAAAGTTAT 5hmC N11↓N9 G

ATTCCAACTACAACTGAACCTAAGG↓CAAAAATCAGCATGAAAATGAATAA 5hmC N11↓N9 G

TTGCAATACCGGAAACGCCATTAAG↓AAGAACAGTAGGAATAATAGGCAAA 5hmC N9↓N10 G

GGGCAAGCTTACGTTCGTAAAGATG↓GCGAATGGGTATTCCTTTCTACCTT 5hmC N9↓N8 G

CTAATCCATCAAAATCCAGTTTAAG↓ATGCGATATTTGATCGCCATCACCT 5hmC N9↓N11 G

GATTTAGCAGTTTCAACACGATAAA↓TTTCGTGACGAAGTTTAGTTATACT 5hmC N11↓N9 G

TTTAATAATCTTTTTCATTTTCTGT↓TCCTCCATAGTTGATAGGGTAATAG 5hmC N9↓N9 G

ATTCCAACTACAACTGAACCTAAGG↓CAAAAATCAGCATGAAAATGAATAA 5hmC N11↓N9 G

___________________________________________________________________

1. T4gt insert was cloned and sequenced from the half SmaI site in pUC19(CCC↓GGG) (not shown). Eco94GmrSD cut sites are indicated by a down arrow, where the upstream T4gt sequences were manually pasted in and regenerated the cleavage junctions. No pUC19 sequence is shown here.
2. These cut sites can be summarized as 5hmC N(17-23) G, where cleavage frequently takes place at the semi-symmetric site 5hmC N(9-11)↓N(8-11)G. When 2Cs (or 2Gs) occur in the cut sites, the number of spacer could be off by 1-2 bases.

**Supplementary Table S2.** Summary of endonuclease activity on T4 DNA and protein expression levels of WT and N-terminus mutants of Eco94GmrSD

| Enzyme | Endonuclease activity | Protein expression level |
| --- | --- | --- |
| WT | +++ Active (100%) | ++ (6xHis) |
| **N-terminus mutants (1 to 6)*** |  |  |
| 1) D217A | +++ Active | + (intein-CBD tag) |
| 2) E228A/D230A | +++ Active | + (intein-CBD tag) |
| 3) D249A | +++ Active | + (intein-CBD tag) |
| 4) E260A/E262A | ++ Partially active (~25%) | + (intein-CBD tag) |
| 5) E271A/E273A | **- Inactive** | ++ (6xHis tag) |
| 6) E278A/K280A | +/- Partially active (<10%) | ++ (6xHis tag) |

* Amino acid residues D217-X(10)-E228-X-D230 or D249-X(10)-E260-X-E262 are located in a putative catalytic site (PD-X-EXE) found in some restriction enzymes. Mutagenesis results indicated that D217, E228, D230, D249, E260, and E262 are not catalytic residues.

**Reference**

1 Webb, B. & Sali, A. Protein structure modeling with MODELLER. *Methods Mol Biol* **1137**, 1-15 (2014).

2 Xu, S. Y. *et al.* Structure Determination and Biochemical Characterization of a Putative HNH Endonuclease from Geobacter metallireducens GS-15. *PLoS One* **8**, e72114 (2013).

3 Bujnicki, J. M., Radlinska, M. & Rychlewski, L. Polyphyletic evolution of type II restriction enzymes revisited: two independent sources of second-hand folds revealed. *Trends Biochem Sci* **26**, 9-11 (2001).

4 Pingoud, A., Fuxreiter, M., Pingoud, V. & Wende, W. Type II restriction endonucleases: structure and mechanism. *Cell Mol Life Sci* **62**, 685-707 (2005).

5 Xu, S. Y. & Gupta, Y. K. Natural zinc ribbon HNH endonucleases and engineered zinc finger nicking endonuclease. *Nucleic acids research* **41**, 378-390 (2013).

6 Bair, C. L., Rifat, D. & Black, L. W. Exclusion of glucosyl-hydroxymethylcytosine DNA containing bacteriophages is overcome by the injected protein inhibitor IPI*. *J Mol Biol* **366**, 779-789 (2007).

7 Jeltsch, A., Alves, J., Maass, G. & Pingoud, A. On the catalytic mechanism of EcoRI and EcoRV. A detailed proposal based on biochemical results, structural data and molecular modelling. *FEBS Lett.* **304**, 4-8 (1992).

8 Xu, S.-y. & Schildkraut, I. Isolation of BamHI variants with reduced cleavage activities. *J. Biol. Chem.* **266**, 4425-4429 (1991).

9 Bair, C. L. & Black, L. W. A type IV modification dependent restriction nuclease that targets glucosylated hydroxymethyl cytosine modified DNAs. *J Mol Biol* **366**, 768-778 (2007).

10 Rifat, D., Wright, N. T., Varney, K. M., Weber, D. J. & Black, L. W. Restriction endonuclease inhibitor IPI* of bacteriophage T4: a novel structure for a dedicated target. *J Mol Biol* **375**, 720-734 (2008).
